# Effect of Chinese Herbal Medicine Therapy on Risks of Overall, Diabetes-Related, and Cardiovascular Diseases-Related Mortalities in Taiwanese Patients With Hereditary Hemolytic Anemias

**DOI:** 10.3389/fphar.2022.891729

**Published:** 2022-05-30

**Authors:** Mu-Lin Chiu, Jian-Shiun Chiou, Chao-Jung Chen, Wen-Miin Liang, Fuu-Jen Tsai, Yang-Chang Wu, Ting-Hsu Lin, Chiu-Chu Liao, Shao-Mei Huang, Chen-Hsing Chou, Cheng-Wen Lin, Te-Mao Li, Yu-Lung Hsu, Ying-Ju Lin

**Affiliations:** ^1^ School of Chinese Medicine, China Medical University, Taichung, Taiwan; ^2^ Genetic Center, Proteomics Core Laboratory, Department of Medical Research, China Medical University Hospital,, Taichung, Taiwan; ^3^ College of Health Care, China Medical University, Taichung, Taiwan; ^4^ Department of Health Services Administration, China Medical University, Taichung, Taiwan; ^5^ Graduate Institute of Integrated Medicine, China Medical University, Taichung, Taiwan; ^6^ Department of Pediatrics, China Medical University Children’s Hospital, Taichung, Taiwan; ^7^ Department of Biotechnology and Bioinformatics, Asia University, Taichung, Taiwan; ^8^ Department of Medical Laboratory Science and Biotechnology, China Medical University, Taichung, Taiwan; ^9^ School of Medicine, China Medical University, Taichung, Taiwan

**Keywords:** hereditary hemolytic anemias, overall mortality, diabetes-related mortality, cardiovascular diseases-related mortality, chinese herbal medicine, network analysis

## Abstract

Hereditary Hemolytic Anemias (HHAs) are a rare but heterogeneous group of erythrocytic diseases, characterized by intrinsic cellular defects due to inherited genetic mutations. We investigated the efficacy of Chinese herbal medicine (CHM) in reducing the overall, diabetes-related, and cardiovascular diseases (CVDs)-related mortalities among patients with HHAs using a nationwide population database. In total, we identified 33,278 patients with HHAs and included 9,222 non-CHM and 9,222 CHM matched pairs after matching. The Cox proportional hazards model was used to compare the risk of mortality between non-CHM and CHM users. The Kaplan-Meier method and log-rank test were used to compare the cumulative incidence mortality between non-CHM and CHM users. The CHM prescription patterns were presented by the association rules and network analyses, respectively. The CHM prescription patterns were presented by the association rules and network analyses, respectively. CHM users showed significant reduced risks for of overall (adjusted hazard ratio [aHR]: 0.67, 95% confidence interval [CI]: 0.61–0.73, *p* < 0.001), diabetes-related (aHR: 0.57, 95% CI: 0.40–0.82, *p* < 0.001), and CVDs-related (aHR: 0.59, 95% CI: 0.49–0.72, *p* < 0.001) mortalities compared with non-CHM users. Two CHM clusters are frequently used to treat Taiwanese patients with HHAs. Cluster 1 is composed of six CHMs: Bei-Mu (BM; *Fritillaria cirrhosa* D.Don), Gan-Cao (GC; *Glycyrrhiza uralensis* Fisch.), Hai-Piao-Xiao (HPX; *Endoconcha Sepiae*), Jie-Geng (JG; *Platycodon grandiflorus* (Jacq.) A.DC.), Yu-Xing-Cao (YXC; *Houttuynia cordata* Thunb.), and Xin-Yi-Qing-Fei-Tang (XYQFT). Cluster 2 is composed of two CHMs, Dang-Gui (DG; *Angelica sinensis* (Oliv.) Diels) and Huang-Qi (HQi; *Astragalus membranaceus* (Fisch.) Bunge). Further randomized clinical trials are essential to evaluate the safety and effectiveness of above CHM products and to eliminate potential biases in the current retrospective study.

## Introduction

Hereditary hemolytic anemias (HHAs) are a rare but heterogeneous group of erythrocytic diseases. HHAs is characterized by the premature destruction of red blood cells (RBCs) due to intrinsic cellular defects caused by inherited genetic mutations (Kim et al., 2017; Shim et al., 2020). HHAs contain three main etiologies, including hemoglobinopathy (e.g., thalassemia and sickle cell disease), RBC membranopathy (e.g., hereditary spherocytosis and hereditary elliptocytosis), and RBC enzymopathy (e.g., glucose-6-phosphate dehydrogenase [G6PD] deficiency, pyruvate kinase [PK] deficiency) (Kim et al., 2017; Shim et al., 2020). Among HHAs, thalassemia is the most common hemoglobinopathy, with a prevalence of 1.67% in the world’s population, with an incidence of 4.4/10,000 newborns afflicted worldwide (Chern et al., 2008; Kadhim et al., 2017). In Taiwan, there has been a high incidence of thalassemia, with approximately 5 and 1.1% for α-thalassemia and β-thalassemia, respectively (Chern et al., 2008).

The primary treatments for HHAs include allogeneic hematopoietic stem cell transplantation (HSCT), splenectomy, and blood transfusion (Vassiliou et al., 2001; Shim et al., 2020; Zaninoni et al., 2020). HSCT results in an 80–95% long term survival rate for some types of HHAs, including thalassemia and sickle cell disease (SCD) (Mentzer, 2000; Vassiliou et al., 2001; Gluckman et al., 2017; Kassim and Sharma, 2017). However, due to the lack of suitable human leukocyte antigen (HLA)-matched donors HSCT is a difficult task. Furthermore, complications of HSCT in HHAs patients are reported, including veno-occlusive disease of the liver, graft-vs-host disease (GVHD), infection, and disease recurrence (Tabbara et al., 2002; Arnaout et al., 2014). Splenomegaly is a typical condition in patients with HHAs and increases erythrocyte destruction (Haley, 2017). Therefore, splenectomy is another effective therapy to decrease erythrocyte catheresis and elevate hemoglobin levels in patients with HHAs (Zaninoni et al., 2020). However, there are many complications after splenectomy, such as infection, thrombosis, cardiovascular diseases (CVDs), pulmonary hypertension, and reduced immune function (Mozos, 2015; Iolascon et al., 2017).

HHAs are chronic and lifelong diseases, and usually require repeated blood transfusions, which may result in iron overload and require iron chelation therapy (Sayani and Kwiatkowski, 2015; Ballas et al., 2018). In Taiwan, thalassemia is responsible for approximately 85% of the HHAs patients and requires frequent blood transfusion and iron chelation therapy. The long-term complications of frequent blood transfusion in patients include hypogonadism (23.1%), diabetes (21.2%), congestive heart failure (18.9%), arrhythmia (17.6%), osteoporosis (17.4%), and liver cirrhosis (16.5%) (Wu et al., 2017). Furthermore, iron overload may lead to serious complications, such as CVDs, diabetes, liver diseases, and infertility (Zaninoni et al., 2020). Among these serious complications, CVDs are major causes of morbidity and mortality in patients with iron overload (Lekawanvijit and Chattipakorn, 2009).

Chinese herbal medicine (CHM) exhibits effective, safe, less toxic, and have few side effects and has been used to treat anemia and blood-related diseases (Liu et al., 2006; Wu et al., 2006; Wang et al., 2008; Gao and Chong, 2012; Chu et al., 2014; Fleischer et al., 2016a; Fleischer et al., 2016b; Fleischer et al., 2017; Chen et al., 2018; Fleischer et al., 2018; Sun et al., 2018; Zhu et al., 2018). Furthermore, due to many complications occurred from the primary treatments for HHAs patients, HHAs patients in Taiwan may also seek for adjunctive therapies to alleviate their anemia symptoms and reduce these side effects. In Taiwan, Chinese herbal medicine (CHM) has been one of the important health care system and has also been widely used in patients with anemia, chronic myeloid leukemia, chronic lymphocytic leukemia, acute myeloid leukemia, and patients with the HSCT treatment (Fleischer et al., 2016a; Fleischer et al., 2016b; Fleischer et al., 2017; Chen et al., 2018; Fleischer et al., 2018). However, there are still limited studies in the CHM effect on patients with HHAs.

To evaluate the prolonged CHM effect on patients with HHAs, we conducted a retrospective cohort study using a nationwide population-based database in Taiwan. We explored the efficacy of CHM usage and the risks of overall, diabetes-related, and CVDs-related mortalities among patients with HHAs.

## Materials and Methods

### Study Subjects

**FIGURE 1:**
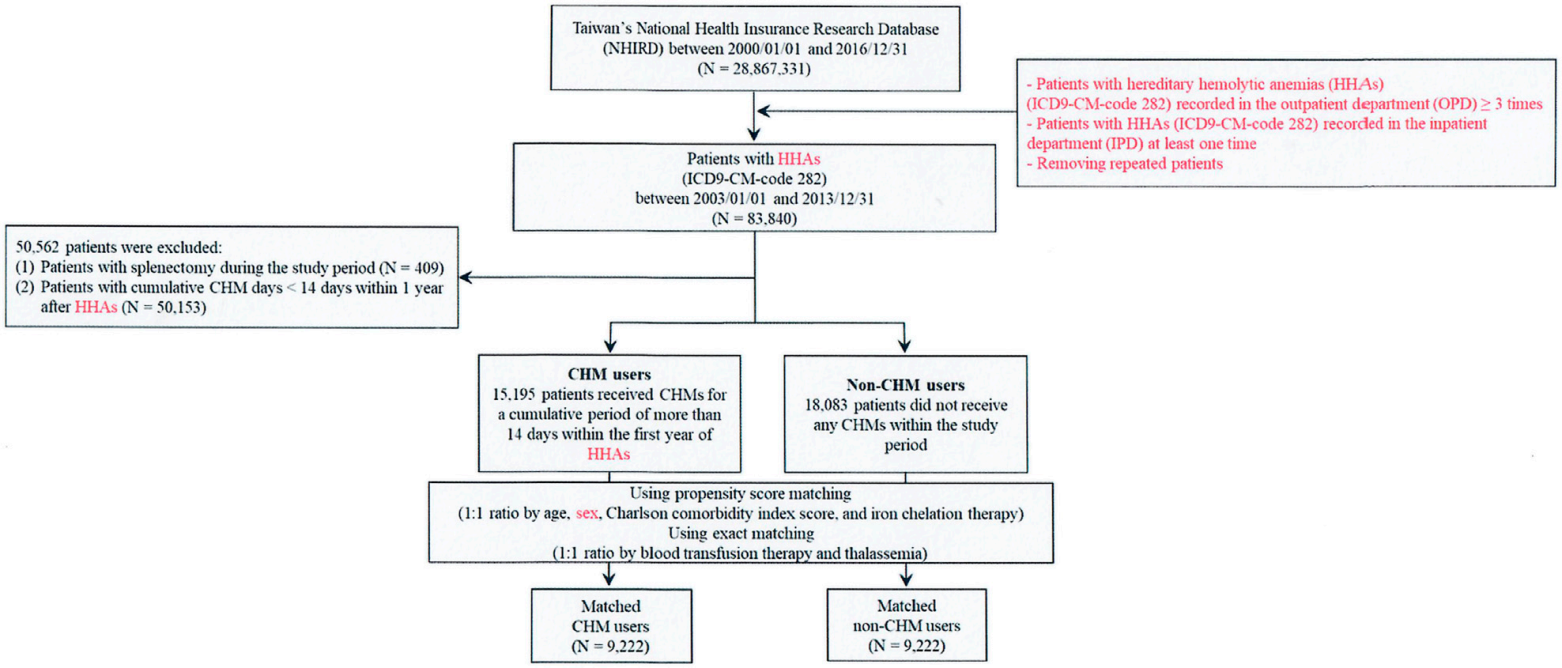
Flowchart of hereditary hemolytic anemias patient enrollment.

In this study, we identified 28,867,331 anonymized beneficiaries from the National Health Insurance Research Database (NHIRD) of Taiwan during Jan. 1, 2000 and Dec. 31, 2016. The International Classification of Disease, 9^th^ Revision, Clinical Modification (ICD-9-CM) code 282 was used to identify patients with hereditary hemolytic anemias (HHAs) during the period between Jan. 1, 2003 and Dec. 31, 2013 (Figure 1).

Patients with HHAs were enrolled with the ICD-9-CM-code 282 recorded in the outpatient department (OPD) ≥ 3 times or the HHAs patients recorded in the inpatient department (IPD) at least one time. After removing the repeated patients, there were resulting in 83,840 patients with HHAs (the ICD-9-CM-code 282). For patients with HHAs, there were 3 main etiologies: hemoglobinopathy, RBC membranopathy, and RBC enzymopathy (Kim et al., 2017; Shim et al., 2020). Patients were excluded (N = 50,562) with the following criteria: 1) patients with less than 14 cumulative days of CHM prescriptions within 1 year following the diagnosis of HHAs (N = 50,153); and 2) patients who underwent splenectomy during the study period (N = 409).

**FIGURE 2:**
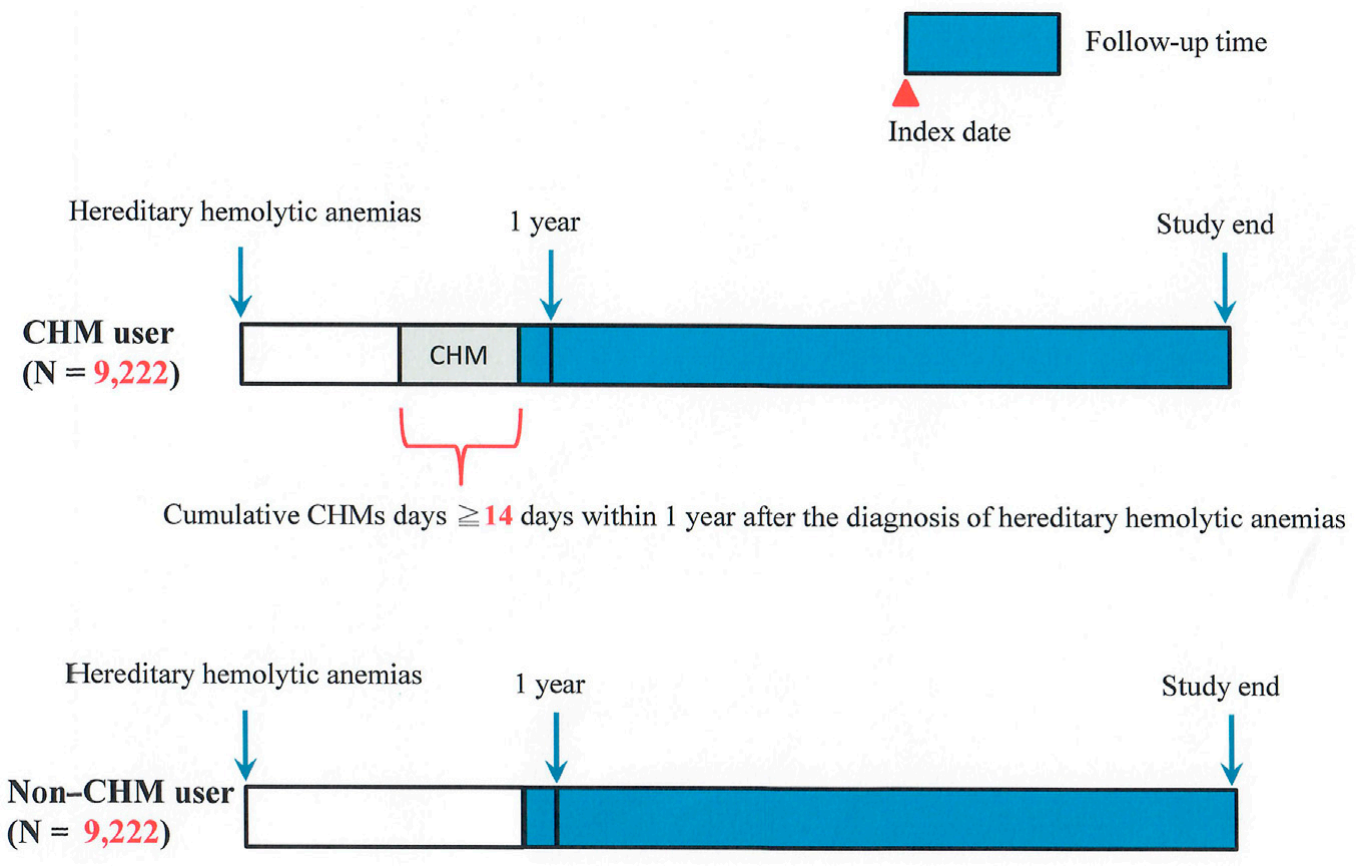
Diagram of follow-up time for patients with HHAs. Abbreviations: HHAs, hereditary hemolytic anemias.

Patients who had more than 14 CHM cumulative days within 1 year after the diagnosis of HHAs were designated as CHM users (N = 15,195) (Figure 1, Figure 2). Patients who had no CHM prescriptions during the study period were designated as non-CHM users (N = 18,083). The non-CHM users and CHM users were matched by age, sex, iron chelation therapy, and Charlson comorbidity index (CCI) at a 1:1 ratio using the propensity score method; and they were also matched by blood transfusion therapy and thalassemia at a 1:1 ratio using the exact matching method. Finally, 9,222 non-CHM users and 9,222 CHM users were identified after matching (Table 1) (Figure 1). The index date is the date with a completion of 14 CHM cumulative days within 1 year after the diagnosis of HHAs (Figure 2). The CHM-users continued to use CHMs during the follow-up period (Supplementary Table S2). The basic characteristics included age, sex, CCI, blood transfusion therapy, iron chelation therapy, and patients with HHAs and thalassemia (Table 1). Comorbidities, blood transfusion therapy, and iron chelation therapy were used within 1 year before or after the diagnosis of HHAs (Table 1). We obtained the ethical approval (CMUH107-REC3-074 [CR1]) of this study from China Medical University and Hospital Research Ethics Committee.

**TABLE 1:** Basic characteristics of patients with hereditary hemolytic anemias (HHAs) in Taiwan.

Characteristics	Total Subjects		*p*-value	Matched Subjects		*p*-value
	CHM users (N = 15,195)	Non-CHM users (N = 18,083)		CHM users (N = 9,222)	Non-CHM users (N = 9,222)	
	N (%)	N (%)		N (%)	N (%)	
Age (years old; Mean ± SD)	37.98 ± 19.49	27.94 ± 27.81	<0.001	36.63 ± 23.07	36.02 ± 25.21	0.087
0≦Age<18	2,393 (15.75%)	8,517 (47.33%)	<0.001	2,385 (25.86%)	2,383 (25.84%)	0.569
18≦Age<40	5,887 (38.75%)	3,472 (19.29%)		2,811 (30.48%)	2,766 (29.99%)	
40≦Age<65	5,407 (35.59%)	3,373 (18.74%)		2,612 (28.32%)	2,693 (29.20%)	
65≦Age	1,504 (9.90%)	2,634 (14.64%)		1,414 (15.33%)	1,380 (14.96%)	
Sex			<0.001			0.572
Male	4,058 (26.87%)	9,616 (54.61%)		3,947 (42.80%)	3,985 (43.21%)	
Female	11,045 (73.13%)	7,991 (45.39%)		5,275 (57.20%)	5,237 (56.79%)	
CCI score (Mean ± SD)	1.27 ± 2.01	1.22 ± 2.27	0.023	1.38 ± 2.10	1.35 ± 2.17	0.427
Iron chelation therapy			0.277			0.922
No	15,134 (99.60%)	17,996 (99.52%)		9,170 (99.44%)	9,169 (99.43%)	
Yes	61 (0.40%)	87 (0.48%)		52 (0.56%)	53 (0.57%)	
Blood transfusion therapy			<0.001			1.000
No	12,724 (83.74%)	14,097 (77.96%)		7,396 (80.20%)	7,396 (80.20%)	
Yes	2,471 (16.26%)	3,986 (22.04%)		1826 (19.80%)	1826 (19.80%)	
Thalassemia			<0.001			1.000
No	1701 (11.19%)	3,224 (17.83%)		1,171 (12.70%)	1,171 (12.70%)	
Yes	13,494 (88.81%)	14,859 (82.17%)		8,051 (87.30%)	8,051 (87.30%)	

*p*-value (*p* < 0.05) was highlighted in bold italic.

HHAs, hereditary hemolytic anemias; CHM, Chinese herbal medicine; N, number; CCI, Charlson comorbidity index; SD, standard deviation; ICD9-CM, the International Classification of Diseases, Ninth Revision, Clinical Modification.

Patients with hereditary hemolytic anemias (ICD9-CM code: 282); patients with thalassemia (ICD9-CM code: 282.4).

Iron chelatiors (ATC code: V03AC01, V03AC02, and V03AC03). Iron chelatiors were used within 1 year before or after the diagnosed date of hereditary hemolytic anemias.

Blood transfusion therapy included procedures (procedure code: 94001, 94005, 93001, 93002, 93003, 93019, 93004, 93007, 93016, and 93023C). Blood transfusion therapy were used within 1 year before or after the diagnosed date of hereditary hemolytic anemias.

Propensity score matching method was performed for age, sex, CCI score, and iron chelation therapy. Exact matching method was performed for blood transfusion therapy and thalassemia.

### Chinese Herbal Medicine

In this study, the licensed Chinese medicine doctors prescribed Chinese herbal medicine for HHAs patients in Taiwan (Supplementary Table S1) (Supplementary Figures S1–S8). The pharmaceutical companies with Good Manufacturing Practice (GMP) certification in Taiwan produced the CHM products, including single herbs and herbal formulas (Li et al., 2018; Cheng et al., 2019a; Tsai et al., 2019a). A single herb includes minerals, the organs of insects or animals, and any part of the plant (e.g. seeds, roots, stems, leaves, flowers, and fruits). The herbal formula is composed of more than two herbs.

### Association Rule

The pattern of CHM prescriptions for patients with HHAs was shown by the association rule (Yang et al., 2013; Tsai et al., 2019a; Cheng et al., 2019b; Tsai et al., 2019b) with SAS version 9.4 (SAS Institute, Cary, NC, United States). The association strength between CHM co-prescriptions (X and Y) were presented by the confidence value (CHM_X→ CHM_Y; %), support value (X) (%), and lift value (Table 5).

### Network Analysis

In this study, the CHM clusters were investigated by network analysis as previously described (Tsai et al., 2019a; Cheng et al., 2019b; Tsai et al., 2019b; Chen CJ. et al., 2020; Tsai et al., 2020) using version 3.7.0 of Cytoscape (https://cytoscape.org/). The red circle, the green circle, and the circle size indicate the herbal formula, a single herb, and the CHM prescription frequency, respectively. The line color and the line size indicate the lift value and the support value between the CHM pairs, respectively. The darker and thicker connection line indicate a higher connection between the CHM pairs.

### Statistical Analysis

Categorical data, including age, sex, blood transfusion therapy, iron chelation therapy, and thalassemia are shown as numbers (percentages). Continuous data, such as age and CCI, are shown as mean ± standard deviation (SD). The χ^2^ test and the Student’s *t*-test were used to analyze the discrepancy between non-CHM users and CHM users for categorical and continuous data, respectively (Table 1).

**FIGURE 3:**
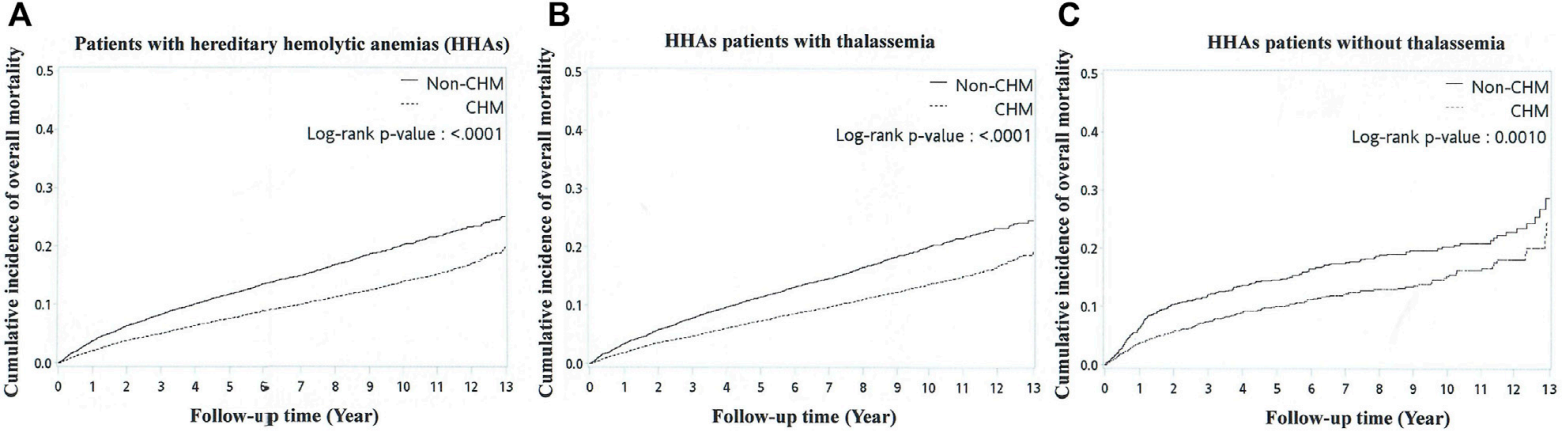
Kaplan‐Meier curves for overall mortality for patients with HHAs **(A)** with/without thalassemia **(B,C)**. Abbreviations: HHAs, hereditary hemolytic anemias.

The crude and adjusted hazard ratios (HRs) were calculated for the risks of overall (Table 2), diabetes-related (Table 3), and CVDs-related mortalities (Table 4) by the Cox proportional hazard model. The overall mortality was adjusted by age, sex, CHM use, CCI, blood transfusion therapy, and iron chelation therapy. The diabetes-related and CVDs-related mortalities were adjusted by age, sex, CHM use, CCI, and blood transfusion therapy. The cumulative incidences of the overall mortality between non-CHM users and CHM users among patients with HHAs and those with and without thalassemia were calculated using the log-rank test and the Kaplan-Meier method (Figure 3). The composition, usage patterns, and frequency of CHM for patients with HHAs are shown (Supplementary Table S1). We used version 9.4 of SAS software (SAS Institute) to analyze all statistical data and *p* < 0.05 was considered as significant.

**TABLE 2:** Risk of overall mortality in patients with hereditary hemolytic anemias (HHAs) in Taiwan.

	Crude			Adjusted		
	HR	95% CI	*p*-value	aHR	95% CI	*p*-value
HHAs patients						
Age (Mean ± SD), per year	1.07	(1.07–1.07)	<0.001	1.05	(1.05–1.05)	<0.001
Sex (Female/male)	0.89	(0.81–0.98)	0.0132	0.75	(0.68–0.82)	<0.001
CHM use	0.67	(0.63–0.71)	<0.001	0.67	(0.61–0.73)	<0.001
CCI score (Mean ± SD), per score	1.42	(1.4–1.44)	<0.001	1.21	(1.19–1.24)	<0.001
Iron chelation therapy (Yes/no)	6.20	(4.75–8.1)	<0.001	1.87	(1.43–2.44)	<0.001
Blood transfusion therapy (Yes/no)	6.67	(6.1–7.29)	<0.001	2.68	(2.44–2.94)	<0.001
HHAs patients with thalassemia						
Age (Mean ± SD), per year	1.07	(1.07–1.07)	<0.001	1.05	(1.05–1.06)	<0.001
Sex (Female/male)	0.85	(0.77–0.93)	<0.001	0.74	(0.67–0.81)	<0.001
CHM use	0.66	(0.62–0.7)	<0.001	0.68	(0.62–0.74)	<0.001
CCI score (Mean ± SD), per score	1.43	(1.4–1.46)	<0.001	1.20	(1.18–1.23)	<0.001
Iron chelation therapy (Yes/no)	5.97	(4.32–8.24)	<0.001	1.74	(1.28–2.37)	<0.001
Blood transfusion therapy (Yes/no)	6.51	(5.91–7.17)	<0.001	2.70	(2.45–2.98)	<0.001
HHAs patients without thalassemia						
Age (Mean ± SD), per year	1.06	(1.06–1.07)	<0.001	1.04	(1.03–1.05)	<0.001
Sex (Female/male)	1.24	(0.97–1.58)	0.084	0.81	(0.63–1.04)	0.100
CHM use	0.71	(0.6–0.83)	<0.001	0.65	(0.5–0.83)	<0.001
CCI score (Mean ± SD), per score	1.39	(1.35–1.44)	<0.001	1.21	(1.16–1.26)	<0.001
Iron chelation therapy (Yes/no)	6.79	(4.55–10.13)	<0.001	2.14	(1.35–3.41)	0.001
Blood transfusion therapy (Yes/no)	7.96	(6.28–10.09)	<0.001	2.71	(2.1–3.49)	<0.001

HHAs, hereditary hemolytic anemias; CHM, Chinese herbal medicine; SD, standard deviation; HR, hazard ratio; aHR, adjusted hazard ratio; CCI, Charlson comorbidity index; 95% CI, 95% confidence interval.

Age (Mean ± SD) and CCI score (Mean ± SD) was expressed as a continuous variable.

Adjusted factors were age, sex, CHM use, CCI score, and usages of iron chelation and blood transfusion therapies.

Usages of therapies were applied within 1 year before or after the diagnosed date of hereditary hemolytic anemias.

Patients with hereditary hemolytic anemias (ICD9-CM code: 282); patients with thalassemia (ICD9-CM code: 282.4).

Significant *p*-values (*p* < 0.05) are highlighted in bold italic font.

**TABLE 3:** Risk of diabetes-related mortality in patients with hereditary hemolytic anemias (HHAs) in Taiwan.

	Crude			Adjusted		
	HR	95% CI	*p*-value	aHR	95% CI	*p*-value
Age (Mean ± SD), per year	1.08	(1.07–1.09)	<0.001	1.06	(1.05–1.07)	<0.001
Sex (Female/male)	1.17	(0.84–1.63)	0.365	0.98	(0.70–1.39)	0.924
CHM use	0.56	(0.40–0.78)	<0.001	0.57	(0.40–0.82)	0.002
CCI score (Mean ± SD), per score	1.47	(1.43–1.51)	<0.001	1.26	(1.21–1.32)	<0.001
Blood transfusion therapy (Yes/no)	6.66	(4.83–9.18)	<0.001	2.38	(1.68–3.37)	<0.001

HHAs, hereditary hemolytic anemias; CHM, Chinese herbal medicine; SD, standard deviation; HR, hazard ratio; aHR, adjusted hazard ratio; CCI, Charlson comorbidity index; 95% CI, 95% confidence interval.

Age (Mean ± SD) and CCI score (Mean ± SD) was expressed as a continuous variable.

Adjusted factors were age, sex, CHM use, CCI score, and blood transfusion therapy.

Blood transfusion therapy was applied within 1 year before or after the diagnosed date of hereditary hemolytic anemias.

Patients with hereditary hemolytic anemias (ICD9-CM code: 282).

Significant *p*-values (*p* < 0.05) are highlighted in bold italic font.

**TABLE 4:** Risk of cardiovascular diseases-related mortality in patients with hereditary hemolytic anemias (HHAs) in Taiwan.

	Crude			Adjusted		
	HR	95% CI	*p*-value	aHR	95% CI	*p*-value
Age (Mean ± SD), per year	1.10	(1.09–1.11)	<0.001	1.09	(1.08–1.1)	<0.001
Sex (Female/male)	0.92	(0.75–1.12)	0.398	0.75	(0.61–0.91)	0.003
CHM use	0.54	(0.45–0.66)	<0.001	0.59	(0.49–0.72)	<0.001
CCI score (Mean ± SD), per score	1.38	(1.35–1.41)	<0.001	1.11	(1.07–1.15)	<0.001
Blood transfusion therapy (Yes/no)	5.94	(4.88–7.22)	<0.001	2.24	(1.83–2.75)	<0.001

HHAs, hereditary hemolytic anemias; CHM, Chinese herbal medicine; SD, standard deviation; HR, hazard ratio; aHR, adjusted hazard ratio; CCI, Charlson comorbidity index; 95% CI, 95% confidence interval.

Age (Mean±SD) and CCI score (Mean±SD) was expressed as a continuous variable.

Adjusted factors were age, sex, CHM use, CCI score, and blood transfusion therapy.

Blood transfusion therapy was applied within one year before or after the diagnosed date of hereditary hemolytic anemias.

Patients with hereditary hemolytic anemias (ICD9-CM code: 282).

Significant *p*-values (*p* < 0.05) are highlighted in bold italic font.

## Results

### Demographic Characteristics of Taiwanese Patients With HHAs

Table 1 presented the demographics among patients with HHAs in Taiwan. In total, 18,083 non-CHM users and 15,195 CHM users were included in this study. There were significant discrepancies among age, sex, CCI score, blood transfusion therapy, and thalassemia between the two groups at baseline (*p* < 0.05; Table 1). After matching, the differences between the 9,222 non-CHM and 9,222 CHM matched pairs were not significant (*p* > 0.05; Table 1). The CHM-users continued to use CHMs during the follow-up period (Supplementary Table S1).

### Risk of Overall Mortality in Taiwanese Patients With HHAs

CHM users had a 33% reduced risk of overall mortality among patients with HHAs in Taiwan compared to non-users after adjusting for age, sex, CCI score, CHM use, and usages of iron chelation and blood transfusion therapies (adjusted hazard ratio [aHR]: 0.67, 95% confidence interval [CI]: 0.61–0.73, *p* < 0.001) (Table 2). Among the patients with HHAs, at least 85% were patients with thalassemia (Table 1). Among HHAs patients with thalassemia, CHM users had a 32% reduced risk of overall mortality (aHR: 0.68, 95% CI: 0.62–0.74, *p* < 0.001) compared with non-users after adjustment (Table 2). Among patients with HHAs but without thalassemia, CHM users presented a 35% reduced risk of overall mortality (aHR: 0.65, 95% CI: 0.50–0.83, *p* < 0.001) compared to non-users after adjustment (Table 2).

An older age was correlated with an elevated risk of overall mortality in patients with HHAs (aHR: 1.05, 95% CI: 1.05–1.05, *p* < 0.001), patients with HHAs and thalassemia (aHR: 1.05, 95% CI: 1.05–1.06, *p* < 0.001), and patients with HHAs but without thalassemia (aHR: 1.04, 95% CI: 1.03–1.05, *p* < 0.001) after adjustment (Table 2). Females had around a 25% reduced risk of overall mortality among patients with HHAs (aHR: 0.75, 95% CI: 0.68–0.82, *p* < 0.001), and patients with HHAs and thalassemia (aHR: 0.74, 95% CI: 0.67–0.81, *p* < 0.001) compared with males after adjustment (Table 2). However, the sex-based difference among patients with HHAs but without thalassemia was not significant. Patients with higher CCI scores presented around a 20% elevated risk of overall mortality among the patients with HHAs (aHR: 1.21, 95% CI: 1.19–1.24, *p* < 0.001), HHAs patients with thalassemia (aHR: 1.20, 95% CI: 1.18–1.23, *p* < 0.001), and patients with HHAs but without thalassemia (aHR: 1.21, 95% CI: 1.16–1.26, *p* < 0.001) after adjustment (Table 2).

Patients who underwent iron chelation therapy had an increased risk of overall mortality than those who did not after adjustment, including patients with HHAs (aHR: 1.87, 95% CI: 1.43–2.44, *p* < 0.001), patients with HHAs and thalassemia (aHR: 1.74, 95% CI: 1.28–2.37, *p* < 0.001), and patients with HHAs but without thalassemia (aHR: 2.14, 95% CI: 1.35–3.41, *p* = 0.001) (Table 2). Patients who received blood transfusion therapy had around a 2.70-fold increased risk in overall mortality than patients who did not after adjustment, including patients with HHAs (aHR: 2.68, 95% CI: 2.44–2.94, *p* < 0.001), patients with HHAs and thalassemia (aHR: 2.70, 95% CI: 2.45–2.98, *p* < 0.001), and patients with HHAs but without thalassemia (aHR: 2.71, 95% CI: 2.10–3.49, *p* < 0.001) (Table 2).

Kaplan‒Meier curves showed the differences in the cumulative incidence of overall mortality between CHM users and non-users among the patients with HHAs and those with and without thalassemia (Figure 3). The cumulative incidences of overall mortality of CHM users were significantly decreased in all three groups compared with non-CHM users (*p* ≤ 0.001, log-rank test).

### Risk of Diabetes-Related Mortality in Taiwanese Patients With HHAs

CHM users had a 43% reduced risk of diabetes-related mortality in HHAs patients (aHR: 0.57, 95% CI: 0.40–0.82, *p* = 0.002) compared with non-users after adjustment (Table 3). An older age also showed an elevated risk of diabetes-related mortality in the patients with HHAs (aHR: 1.06, 95% CI: 1.05–1.07, *p* < 0.001) (Table 3). However, the sex-based difference in patients with HHAs was not significant. Patients with higher CCI scores presented a 1.26-fold increase in risk of diabetes-related mortality in patients with HHAs (aHR, 1.26; 95% CI: 1.21–1.32, *p* < 0.001) after adjustment (Table 3). Patients who received blood transfusion therapy presented a 2.38-fold higher risk of diabetes-related mortality (aHR: 2.38, 95% CI: 1.68–3.37, *p* < 0.001) than those who did not in patients with HHAs after adjustment (Table 3).

### Risk of CVDs-Related Mortality in Taiwanese Patients With HHAs

CHM users showed around a 40% reduced risk of CVDs-related mortality in patients with HHAs (aHR: 0.59, 95% CI: 0.49–0.72, *p* < 0.001) compared with non-users after adjustment (Table 4). Higher age also showed an elevated CVDs-related mortality risk in patients with HHAs (aHR: 1.09, 95% CI: 1.08–1.10, *p* < 0.001) after adjustment (Table 4). Females had a 25% reduced risk of CVDs-related mortality in patients with HHAs (aHR, 0.75; 95% CI, 0.61–0.91; *p* = 0.003) compared to males after adjustment (Table 4). Patients with higher CCI scores had a 1.11-fold increase in risk of CVDs-related mortality in the patients with HHAs (aHR, 1.11; 95% CI: 1.07–1.15, *p* < 0.001) after adjustment (Table 4). Patients with HHAs who received blood transfusion therapy presented a 2.24-fold increase in risk of CVDs-related mortality (aHR: 2.24, 95% CI: 1.83–2.75, *p* < 0.001) than those who did not after adjustment (Table 4).

### CHM Prescription Pattern in Taiwanese Patients With HHAs

Supplementary Table S1 presented the CHM composition and prescription frequency among Taiwanese patients with HHAs. The most frequently used herbal formula was Xin-Yi-Qing-Fei-Tang (XYQFT). The most commonly used single herbs included Gan-Cao (GC; *Glycyrrhiza uralensis* Fisch.), followed by Jie-Geng (JG; *Platycodon grandiflorus* (Jacq.) A.DC.), Bei-Mu (BM; *Fritillaria cirrhosa* D.Don), Huang-Qi (HQi; *Astragalus membranaceus* (Fisch.) Bunge), Yu-Xing-Cao (YXC; *Houttuynia cordata* Thunb.), Hai-Piao-Xiao (HPX; *Endoconcha Sepiae*), and Dang-Gui (DG; *Angelica sinensis* (Oliv.) Diels).

Table 5 presented the most frequently used CHM co-prescriptions among patients with HHAs in Taiwan. There were 9,222 CHM users and 378,859 prescriptions during the study period. According to the association rule, higher support value, confidence value, and lift value are corresponding to stronger correlations between CHM co-prescriptions. As a results, the most commonly prescribed CHM co-prescriptions were Bei-Mu (BM; *Fritillaria cirrhosa* D.Don)→Jie-Geng (JG; *Platycodon grandiflorus* (Jacq.) A.DC.) (First co-prescription frequency: 4,239, support: 1.119%, confidence: 19.124%, lift: 3.214), followed by Hai-Piao-Xiao (HPX; *Endoconcha Sepiae*)→Bei-Mu (BM; *Fritillaria cirrhosa* D.Don) (Second co-prescription frequency: 3,715, support: 0.981%, confidence: 33.075%, lift: 5.653), and Jie-Geng (JG; *Platycodon grandiflorus* (Jacq.) A.DC.)→Gan-Cao (GC; *Glycyrrhiza uralensis* Fisch.) (Third co-prescription frequency: 3,615, support: 0.954%, confidence: 16.037%, lift: 2.443) (Table 5).

**TABLE 5:** Most commonly used pairs of CHM products for patients with hereditary hemolytic anemias in Taiwan.

CHM products (LHS, X)	Chinese name	Frequency of prescriptions of X product		CHM products (RHS, Y)	Chinese name	Frequency of prescriptions of Y product	Frequency of prescriptions of X and Y products	Support (X) (%)	Confidence (X →Y) (%)	Lift
Bei-Mu (BM; Fritillaria cirrhosa D.Don)	貝母	22,166	→	Jie-Geng (JG; Platycodon grandiflorus (Jacq.) A.DC.)	桔梗	22,542	4,239	1.119	19.124	3.214
Hai-Piao-Xiao (HPX; Endoconcha Sepiae)	海螵蛸	11,232	→	Bei-Mu (BM; Fritillaria cirrhosa D.Don)	貝母	22,166	3,715	0.981	33.075	5.653
Jie-Geng (JG; Platycodon grandiflorus (Jacq.) A.DC.)	桔梗	22,542	→	Gan-Cao (GC; Glycyrrhiza uralensis Fisch.)	甘草	24,870	3,615	0.954	16.037	2.443
Yu-Xing-Cao (YXC; Houttuynia cordata Thunb.)	魚腥草	15,185	→	Xin-Yi-Qing-Fei-Tang (XYQFT)	辛夷清肺湯	22,590	3,584	0.946	23.602	3.958
Dang-Gui (DG; Angelica sinensis (Oliv.) Diels))	當歸	9,518	→	Huang-Qi (HQi; Astragalus membranaceus (Fisch.) Bunge)	黃耆	18,713	3,372	0.890	35.428	7.173

CHM, Chinese herbal medicine; LHS, left-hand-side; RHS, right-hand-side.

Total prescriptions = 378,859.

Support (X) (%) = Frequency of prescriptions of X and Y products/total prescriptions x 100%.

Confidence (X →Y) (%) = Frequency of prescriptions of X and Y products/Frequency of prescriptions of X product x 100%.

Lift = Confidence (X →Y) (%)/P (Y) (%).

P (Y) (%) = Frequency of prescriptions of Y product/total prescriptions x 100%.

**FIGURE 4:**
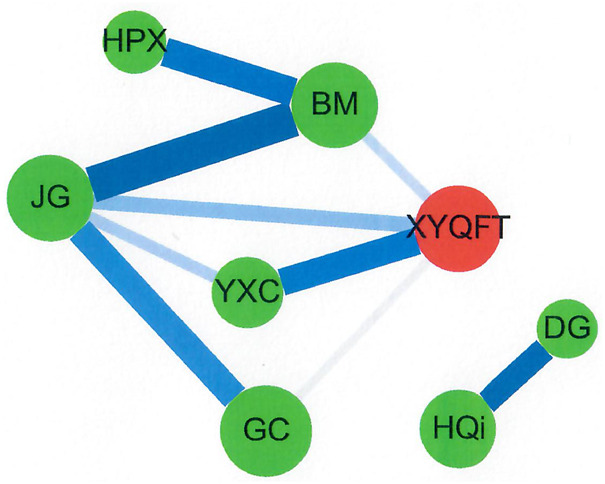
CHM network analysis in patients with HHAs. Herbal formula is shown as the red circle, and single herb is expressed as the green circle. The circle size indicates prescription frequency of the CHM. The line size and color represent the support value and lift value between paired CHM products, respectively. The thicker and darker connection line shows the stronger strength of connection between the paired CHM products. Abbreviations: HHAs, hereditary hemolytic anemias; CHM, Chinese herbal medicine.

The network analysis presented the CHM prescription patterns among patients with HHAs in Taiwan (Figure 4). Two clusters with eight CHMs are significant for these patients. In cluster 1, Gan-Cao (GC; *Glycyrrhiza uralensis* Fisch.), Jie-Geng (JG; *Platycodon grandiflorus* (Jacq.) A.DC.), Bei-Mu (BM; *Fritillaria cirrhosa* D.Don), Yu-Xing-Cao (YXC; *Houttuynia cordata* Thunb.), Hai-Piao-Xiao (HPX; *Endoconcha Sepiae*), and XYQFT are important CHMs. In cluster 2, Huang-Qi (HQi; *Astragalus membranaceus* (Fisch.) Bunge) and Dang-Gui (DG; *Angelica sinensis* (Oliv.) Diels) are important CHMs.

## Discussion

The effectiveness, safety, less toxicity, and few side effects of Chinese herbal medicine (CHM) in treating anemia and blood-related diseases are reported (Liu et al., 2006; Wu et al., 2006; Wang et al., 2008; Gao and Chong, 2012; Chu et al., 2014; Fleischer et al., 2016a; Fleischer et al., 2016b; Fleischer et al., 2017; Chen et al., 2018; Fleischer et al., 2018; Sun et al., 2018; Zhu et al., 2018). However, the long-term effect of CHM on patients with hereditary hemolytic anemias (HHAs) remain unclear. The aim of this retrospective cohort study was to investigate the effect of CHM on the risks of overall, diabetes-related, and CVDs-related mortalities in patients with HHAs using a nationwide population database in Taiwan. In this study, at least 85% of the patients with HHAs were diagnosed with thalassemia. Patients with CHM usage had significantly reduced risks of overall, diabetes-related, and CVDs-related mortalities, respectively, when compared with non-users. We also identified that two CHM clusters with eight CHMs are important for patients with HHAs. Our findings suggested that the protective effect of CHMs against the risks of overall, diabetes-related, and CVDs-related mortalities was observed in patients with HHAs and may implicate their clinical potential as an adjunctive therapy.

Our study results supported previous studies that showing that CHMs are effective, safe, less toxic, and have few side effects in treating anemia and blood-related diseases (Liu et al., 2006; Wu et al., 2006; Wang et al., 2008; Gao and Chong, 2012; Chu et al., 2014; Fleischer et al., 2016a; Fleischer et al., 2016b; Cheng et al., 2016; Fleischer et al., 2017; Chen et al., 2018; Fleischer et al., 2018; Sun et al., 2018; Zhu et al., 2018). Zhang et al. reported that a complex Chinese medicine, Yisui Shengxue Granule (YSSXG) may increase the relative mRNA expression level of the gamma-globin gene in the human K562 myelogenous cells *in vitro* (Zhang and Wu, 2008). For patients with thalassemia, Wu et al., reported that the YSSXG may increase the blood levels of hemoglobin (Hb) and red blood cell (RBC; erythrocytes) in patients with thalassemia (Wu et al., 2006). Chu et al. reported that this YSSXG Chinese medicine may contribute to these patients with thalassemia via increasing the relative mRNA expression levels of globin genes and enhancing the antioxidant activities in erythrocytes (Chu et al., 2014). Cheng et al., reported that YSSXG Chinese medicine may also increase proliferation of hematopoietic stem cells (Cheng et al., 2016). While, Liu et al. suggested that YSSXG Chinese medicine may have a protective effect against thalassemia via inhibiting the formation of alpha-globin-cytotoxic precipitates in erythrocytes by upregulating GATA-1 transcription factor and alpha-hemoglobin stabilizing protein (AHSP) (Liu et al., 2006). For patients with hemoglobin H disease, Wang et al., reported that this YSSXG Chinese medicine may also increase the blood levels of Hb and RBC via enhancing the antioxidant activities in erythrocytes (Wang et al., 2008; Wang et al., 2012). Furthermore, natural compounds from Chinese herbs have also been reported for the benefit effects for the antioxidant activity against iron overload, erythroid differentiation, hemoglobin expression, and the colony formation of hematopoietic stem cells (Thephinlap et al., 2007; Ma et al., 2013; Lin et al., 2015; Ren et al., 2016; Liu et al., 2020).

In the present study, we found two main clusters with eight CHMs are important for Taiwanese patients with HHAs. Cluster 1 included XYQFT, Gan-Cao (GC; *Glycyrrhiza uralensis* Fisch.), Jie-Geng (JG; *Platycodon grandiflorus* (Jacq.) A.DC.), Bei-Mu (BM; *Fritillaria cirrhosa* D.Don), Yu-Xing-Cao (YXC; *Houttuynia cordata* Thunb.), and Hai-Piao-Xiao (HPX; *Endoconcha Sepiae*). Cluster 2 included Huang-Qi (HQi; *Astragalus membranaceus* (Fisch.) Bunge) and Dang-Gui (DG; *Angelica sinensis* (Oliv.) Diels). In cluster 1, Xin-Yi-Qing-Fei-Tang (XYQFT) was the most commonly used herbal formula for patients with HHAs in Taiwan. The therapeutic benefits for HHAs in the ingredients of XYQFT may as follows: Zhi-Zi (ZZ; *Gardenia jasminoides* J.Ellis) shows the antithrombotic and antioxidant activities (Pham et al., 2000; Zhang et al., 2013). Gan-Cao (GC; *Glycyrrhiza uralensis* Fisch.) could nourish blood deficiency and exhibit antioxidant activities (Liu YY. et al., 2019; Lin et al., 2019). Huang-Qin (HQin; *Scutellaria baicalensis* Georgi) might also exhibits the antithrombotic, antioxidant, and anti-inflammatory activities (Huang et al., 2006; Ku and Bae, 2014). Sheng-Ma (SM; *Cimicifuga heracleifolia* Kom.) also shows the antioxidant activity (Li et al., 2012).

For the single herbs in cluster 1, Gan-Cao (GC; *Glycyrrhiza uralensis* Fisch.; family Fabaceae) is the most frequently used single herb for Taiwanese patients with HHAs. The combination of Gan-Cao (GC; *Glycyrrhiza uralensis* Fisch.), Huanglian, and Huangqin effectively reduces the osmotic fragility of erythrocytes for G6PD deficiency, the most prevalent disorder of RBC metabolism affecting 400 million people worldwide (Lin et al., 1998; Lippi and Mattiuzzi, 2020). Our HHAs patients with CHM usage had significantly reduced risks of overall, diabetes-related, and CVDs-related mortalities, respectively. Jie-Geng (JG; *Platycodon grandiflorus* (Jacq.) A.DC.; family Campanulaceae) has been used as a remedy against CVDs, hypertension, hyperlipidemia, and diabetes (Lin et al., 2017). Jie-Geng (JG; *Platycodon grandiflorus* (Jacq.) A.DC.) extract prevents cardiomyocyte apoptosis by inhibiting the Ang II-induced IGF-IIR signaling pathway (Lin et al., 2017). Jie-Geng (JG; *Platycodon grandiflorus* (Jacq.) A.DC.) contains flavonoids, saponins, and platycodigenin and possesses antioxidant and anti-inflammatory effects (Fu et al., 2009). Platycosides from Jie-Geng (JG; *Platycodon grandiflorus* (Jacq.) A.DC.) regulate cholesterol-lowering activities (Nyakudya et al., 2014). Bei-Mu (BM; *Fritillaria cirrhosa* D.Don; family Liliaceae) exhibits analgesic, antioxidative, and anti-inflammatory effects (Chen T. et al., 2020). Yu-Xing-Cao (YXC; *Houttuynia cordata* Thunb.; family Saururaceae) possesses antioxidant, anti-inflammatory, and anti-diabetes effects (Kang et al., 2013; Kumar et al., 2014; Woranam et al., 2020). Hai-Piao-Xiao (HPX; *Endoconcha Sepiae*; family Sepiidae; cuttlefish bone) combined with other Chinese herbs are associated with the lower risk of stroke among vertigo patients (Tsai et al., 2016).

In cluster 2, patients with HHAs in Taiwan frequently used the combination of Huang-Qi (HQi; *Astragalus membranaceus* (Fisch.) Bunge) and Dang-Gui (DG; *Angelica sinensis* (Oliv.) Diels). This combination is often used to treat iron-deficiency anemia by improving hemoglobin levels and increasing iron levels through ferritin synthesis (Huang et al., 2016). The synergistic effect of Huang-Qi (HQi; *Astragalus membranaceus* (Fisch.) Bunge) and Dang-Gui (DG; *Angelica sinensis* (Oliv.) Diels) could also balance T lymphocytes, accelerate the recovery of hematopoietic stem cells, restore the balance of the T cell immune response, and inhibit the immune attack-induced apoptosis of bone marrow cells (Liu J. et al., 2019). Huang-Qi (HQi; *Astragalus membranaceus* (Fisch.) Bunge) alone may recover the function of megekaryocyte hematopoiesis and increase serum megakaryocyte colony-stimulating activity in anemic mice (Zhu and Zhu, 2001). Astragalus polyasccharides extracted from Huang-Qi (HQi; *Astragalus membranaceus* (Fisch.) Bunge) could stimulate the liver to secrete hepcidin to activate p38 mitogen-activated protein kinase and IL-6 to lower the iron load in mice (Ren et al., 2016). Angelica sinensis polysaccharide (ASP) from the Dang-Gui (DG; *Angelica sinensis* (Oliv.) Diels) could revive the function of hematopoietic stem cells and prevent mitochondrial apoptosis by inhibiting T cell immune abnormality (Chen Z. et al., 2020). ASP also inhibited the activation of NF-κB p65 through the IκB kinases-IκBα pathway, thereby decreasing the production of TNF-α and IL-6, which is known to suppress erythropoiesis (Wang et al., 2017).

Patients with HHAs who used CHMs as an adjunct therapy showed reduced overall, diabetes-related, and CVDs-related mortalities compared with non-users in our database analysis. CHM therapy may exhibit protective effects against HHAs. We provide a comprehensive list of CHM products, including one herbal formula (XYQFT) and seven single herbs (Gan-Cao (GC; *Glycyrrhiza uralensis* Fisch.), Jie-Geng (JG; *Platycodon grandiflorus* (Jacq.) A.DC.), Bei-Mu (BM; *Fritillaria cirrhosa* D.Don), Huang-Qi (HQi; *Astragalus membranaceus* (Fisch.) Bunge), Yu-Xing-Cao (YXC; *Houttuynia cordata* Thunb.), Hai-Piao-Xiao (HPX; *Endoconcha Sepiae*), and Dang-Gui (DG; *Angelica sinensis* (Oliv.) Diels)), contributing to the potential protective clinical effect to provide an excellent rationale for further double-blind, placebo-controlled clinical trials for patients with HHAs. Further studies are required to clarify the mechanism of the aforementioned CHMs in treating patients with HHAs.

## Data Availability

The data analyzed in this study is subject to the following licenses/restrictions: The datasets presented in this article are not readily available because Only a limited number of databases allowed access to raw data from the Taiwanese NHIRD database. Requests to access the datasets should be directed to Y-JL, yjlin.kath@gmail.com. Requests to access these datasets should be directed to Y-JL, yjlin.kath@gmail.com.
