# Supplementary material for: Effect of Chinese Herbal Medicine Therapy on Risks of Overall, Diabetes-Related, and Cardiovascular Diseases-Related Mortalities in Taiwanese Patients With Hereditary Hemolytic Anemias
Source: Front Pharmacol. 2022 May 30;13:891729. doi: 10.3389/fphar.2022.891729 (PMC9195623; doi:10.3389/fphar.2022.891729)
Supplement: Supplementary file 1 [file DataSheet1.docx]

**Supplementary Information**

Supplementary Text

Figures S1-S8

Tables S1-S2

**Supplementary Text**

**Figure S1.** LC-MS/MS analysis of chemical markers and the extracts of Xin-Yi-Qing-Fei-Tang (XYQFT). (A) Extracted ion chromatogram (EIC) of standards of licochalcone a and glabridin. (B) Base peak chromatogram (BPC) and EIC of the extracts of XYQFT.

**Figure S2.** LC-MS/MS analysis of chemical markers and the extracts of Gan-Cao (GC; *Glycyrrhiza uralensis* Fisch.). (A) Extracted ion chromatogram (EIC) of standards of licochalcone a and glabridin. (B) Base peak chromatogram (BPC) and EIC of the extracts of Gan-Cao (GC; *Glycyrrhiza uralensis Fisch*.).

**Figure S3.** LC-MS/MS analysis of chemical markers and the extracts of Jie-Geng (JG; *Platycodon grandiflorus* (Jacq.) A.DC.). (A) Extracted ion chromatogram (EIC) of standards of platycodin D and deapioplatycodin D. (B) Base peak chromatogram (BPC) and EIC of the extracts of Jie-Geng (JG; *Platycodon grandiflorus* (Jacq.) A.DC.).

**Figure S4.** LC-MS/MS analysis of chemical markers and the extracts of Bei-Mu (BM; *Fritillaria cirrhosa* D.Don). (A) Extracted ion chromatogram (EIC) of standards of sipeimine and peimine. (B) Base peak chromatogram (BPC) and EIC of the extracts of Bei-Mu (BM; *Fritillaria cirrhosa* D.Don).

**Figure S5.** LC-MS/MS analysis of chemical markers and the extracts of Huang-Qi (HQi; *Astragalus membranaceus* (Fisch.) Bunge). (A) Extracted ion chromatogram (EIC) of standards of calycosin and astragaloside IV. (B) Base peak chromatogram (BPC) and EIC of the extracts of Huang-Qi (HQi; *Astragalus membranaceus* (Fisch.) Bunge).

**Figure S6.** LC-MS/MS analysis of chemical markers and the extracts of Yu-Xing-Cao (YXC; *Houttuynia cordata* Thunb.). (A) Extracted ion chromatogram (EIC) of standards of quercitrin and succinic acid. (B) Base peak chromatogram (BPC) and EIC of the extracts of Yu-Xing-Cao (YXC; *Houttuynia cordata* Thunb.).

**Figure S7.** LC-MS/MS analysis of chemical markers and the extracts of Dang-Gui (DG; *Angelica sinensis* (Oliv.) Diels). (A) Extracted ion chromatogram (EIC) of standards of ligustilide and 3-n-butylphthalide. (B) Base peak chromatogram (BPC) and EIC of the extracts of Dang-Gui (DG; *Angelica sinensis* (Oliv.) Diels).

**Figure S8.** Certificate of analysis for Hai-Piao-Xiao (HPX; *Endoconcha Sepiae*).

**TABLE S1 |** Composition of the most commonly used herbal formulas and single herbs for patients with hereditary hemolytic anemias in Taiwan.

**TABLE S2 |** Frequency distribution of the cumulative CHM treatment days during the study period among patients with hereditary hemolytic anemias in Taiwan.

**Figure S1. A**

**
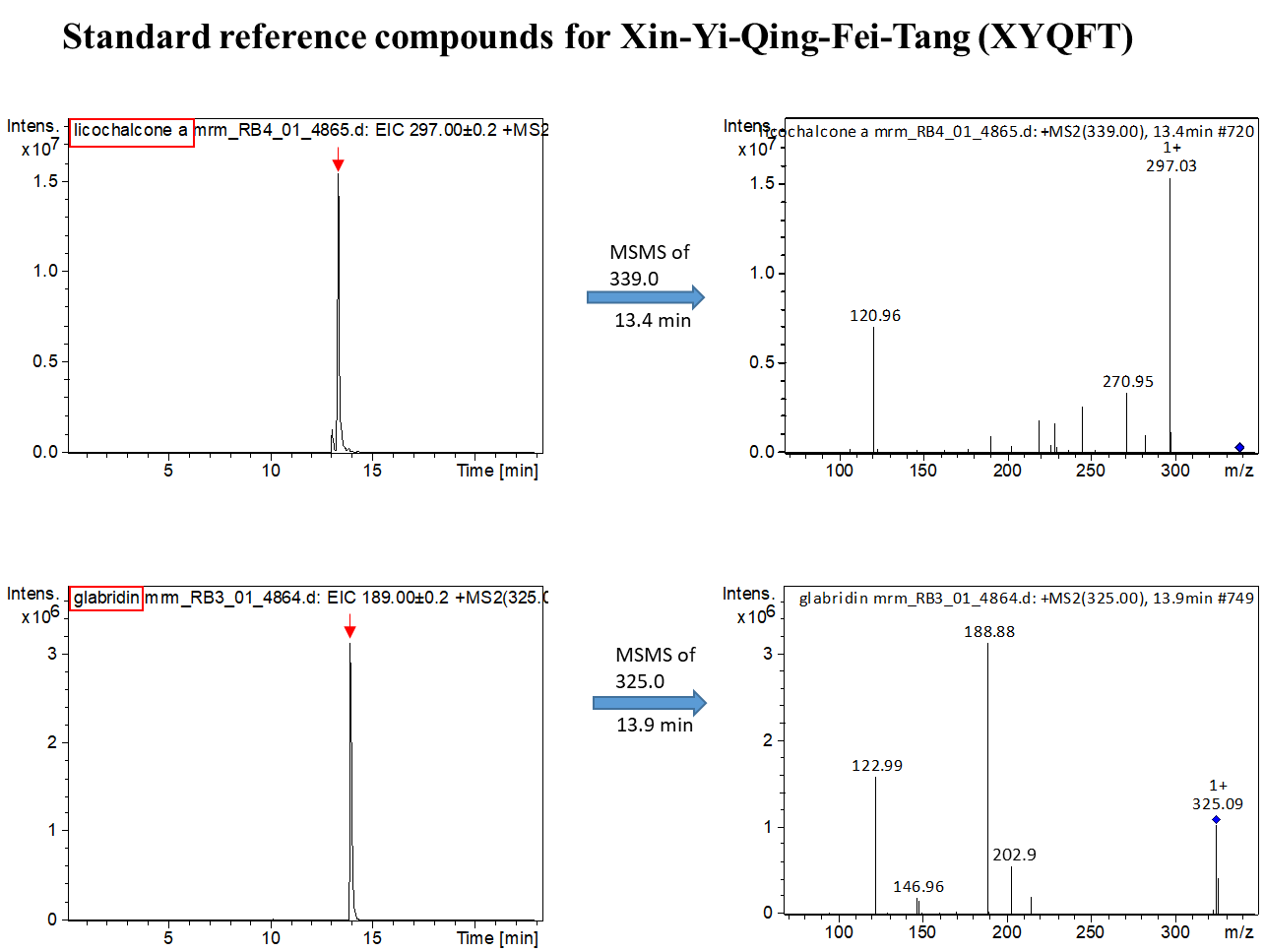
**

**Figure S1. B**

**
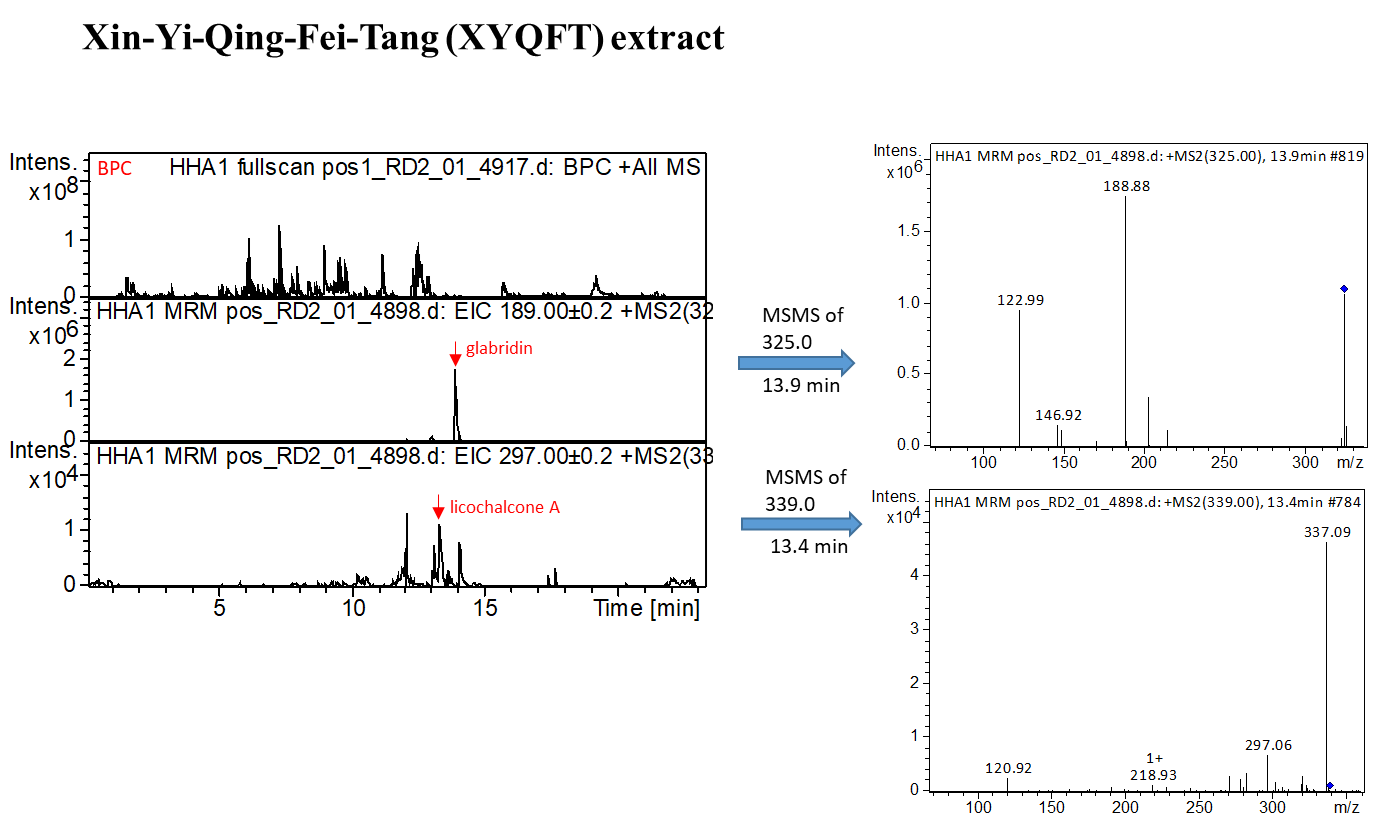
**

**Figure S2. A**

**
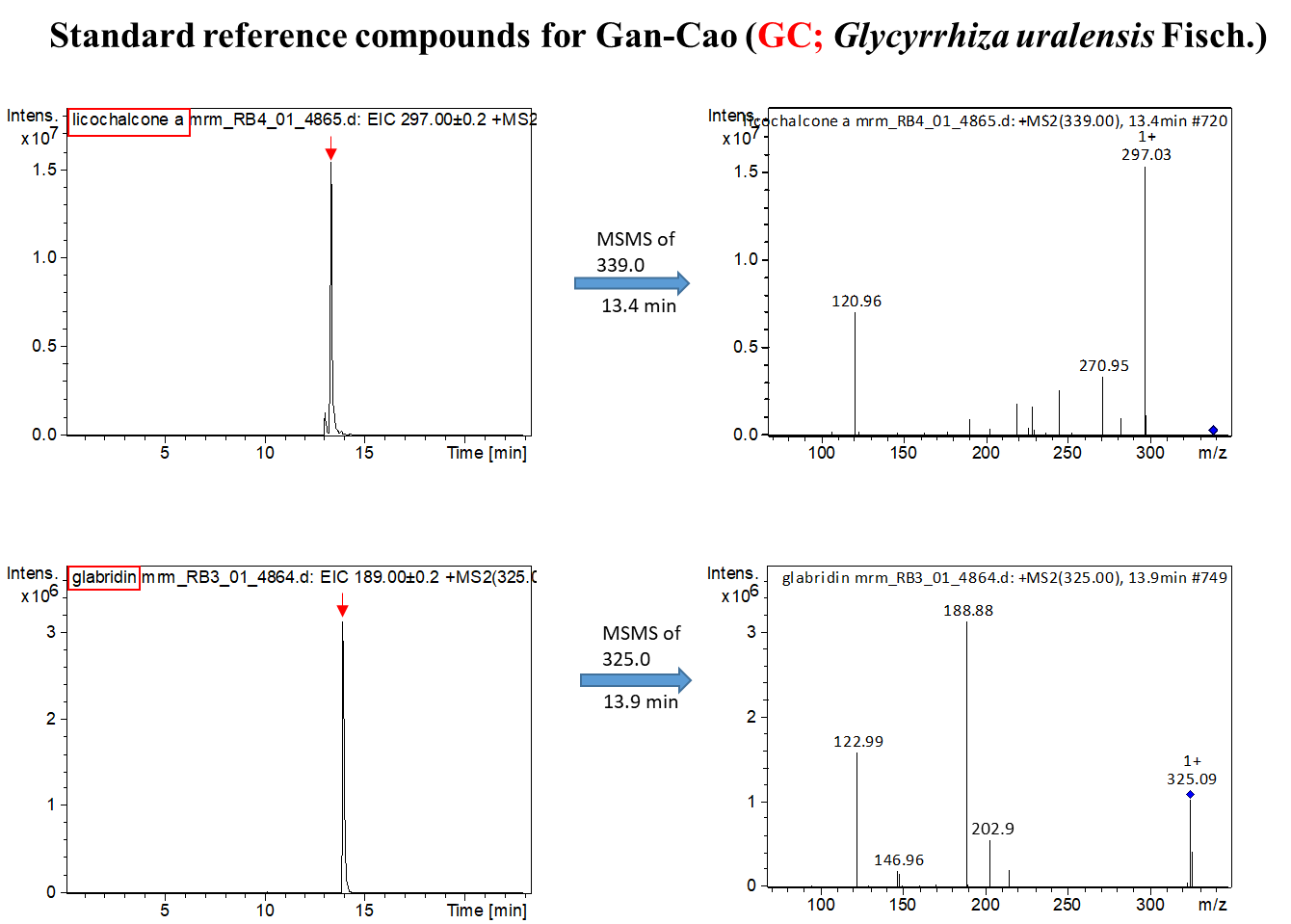
**

**Figure S2. B**

**
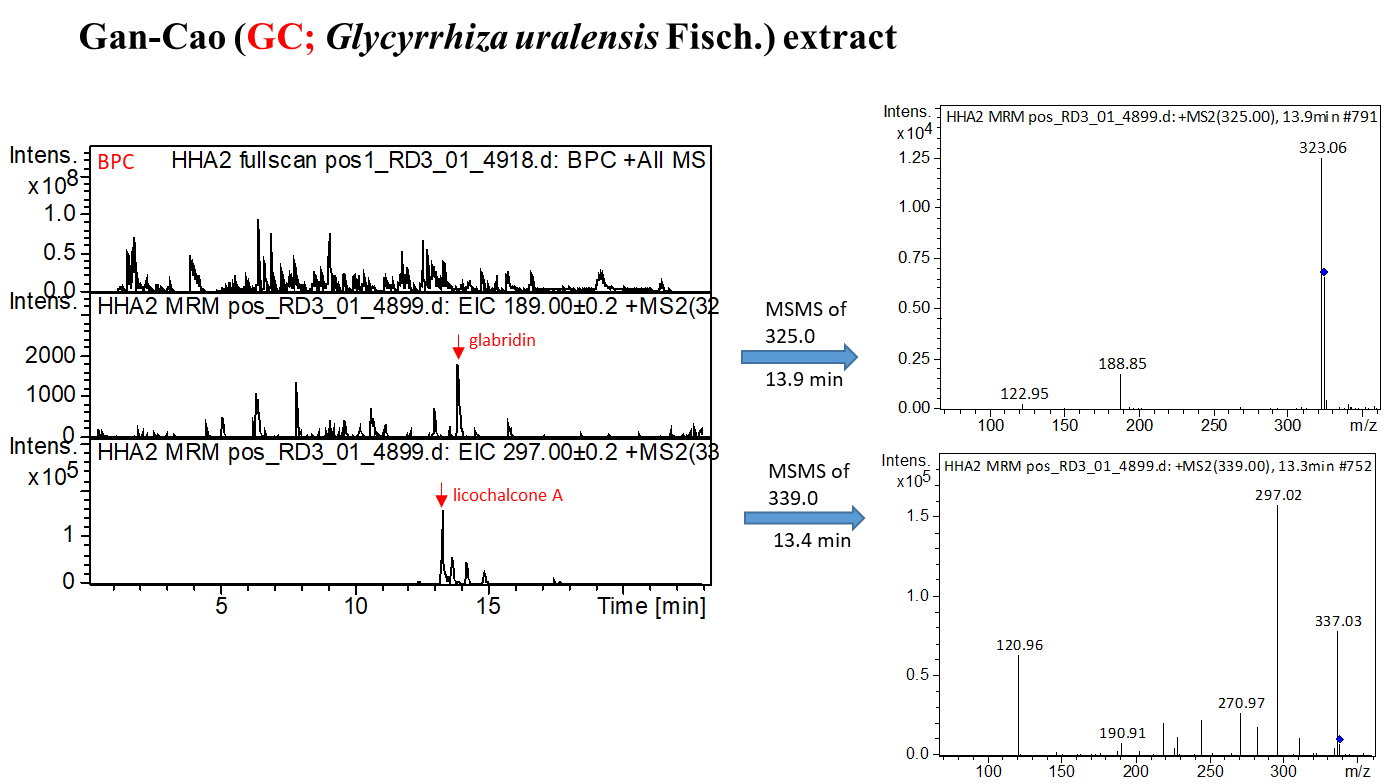
**

**Figure S3. A**

**
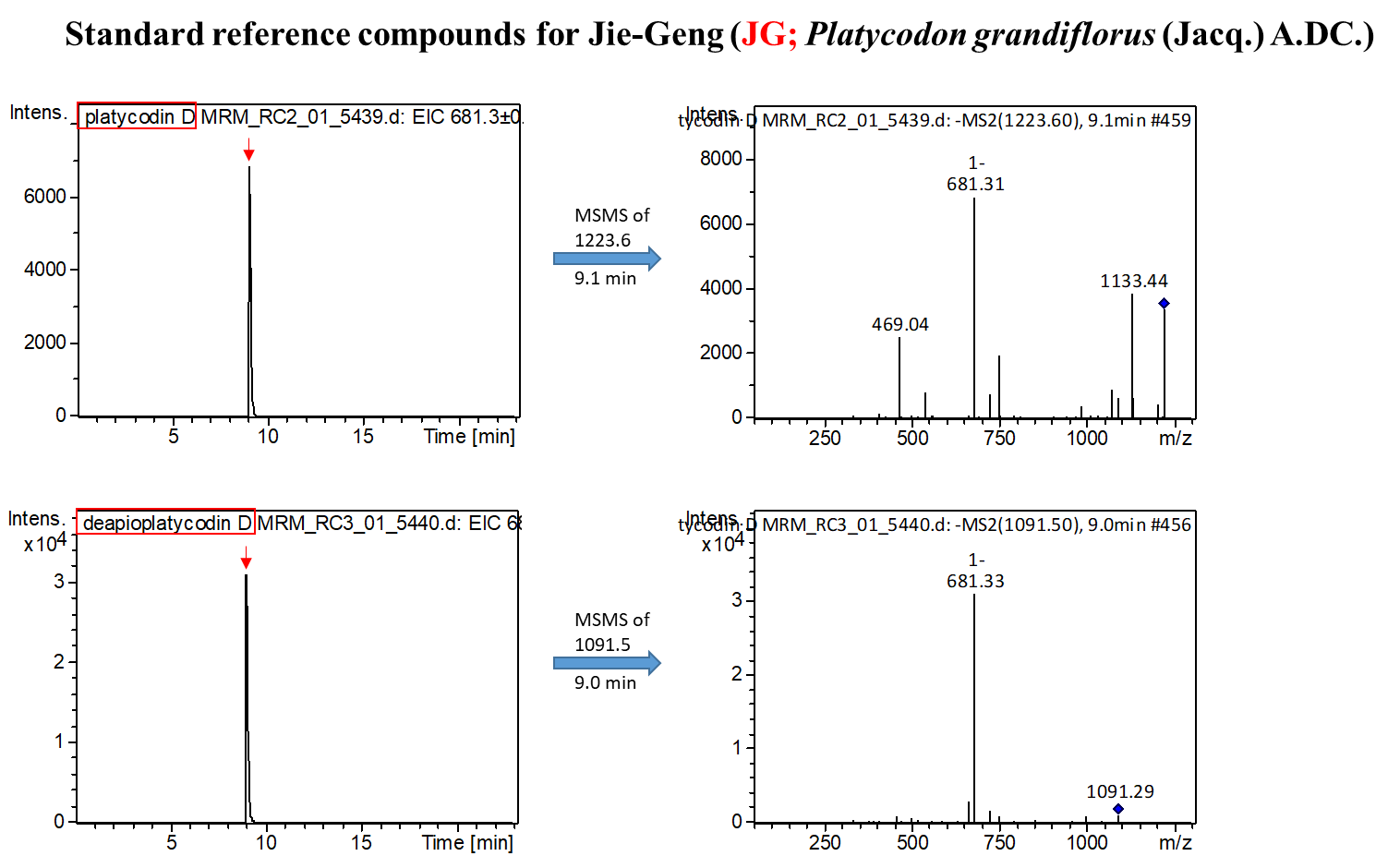
**

**Figure S3. B**


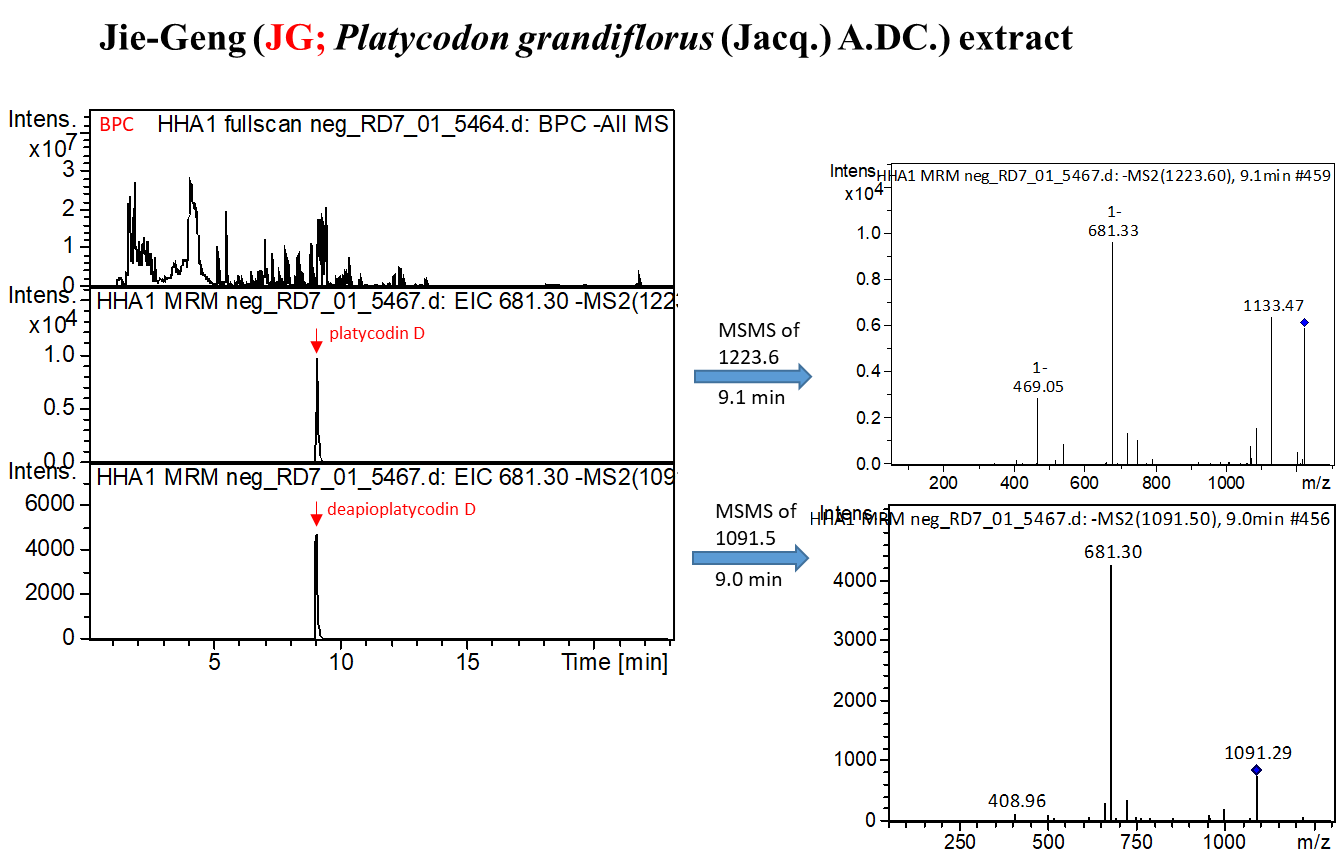


**Figure S4. A**

**
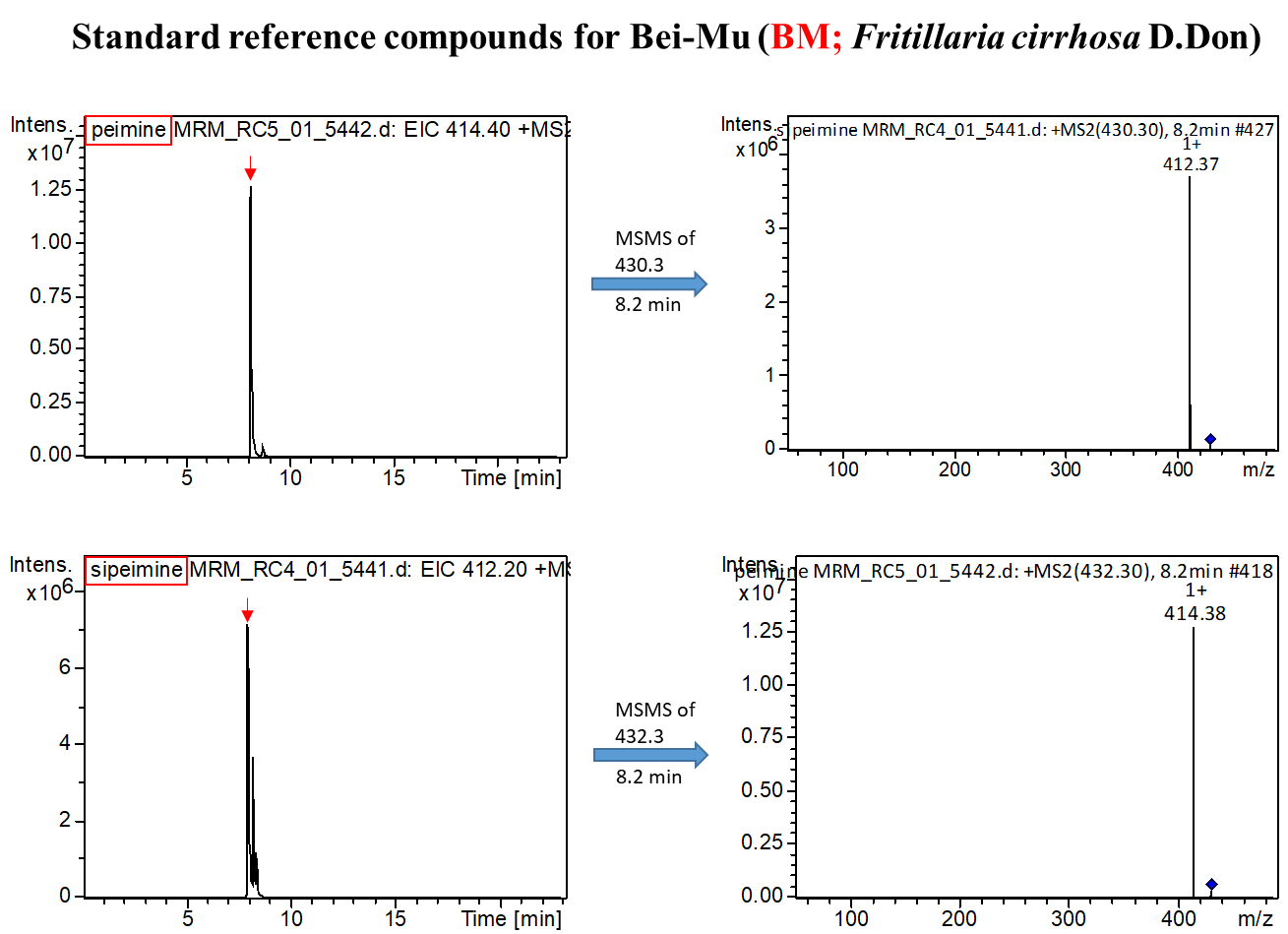
**

**Figure S4. B**

**
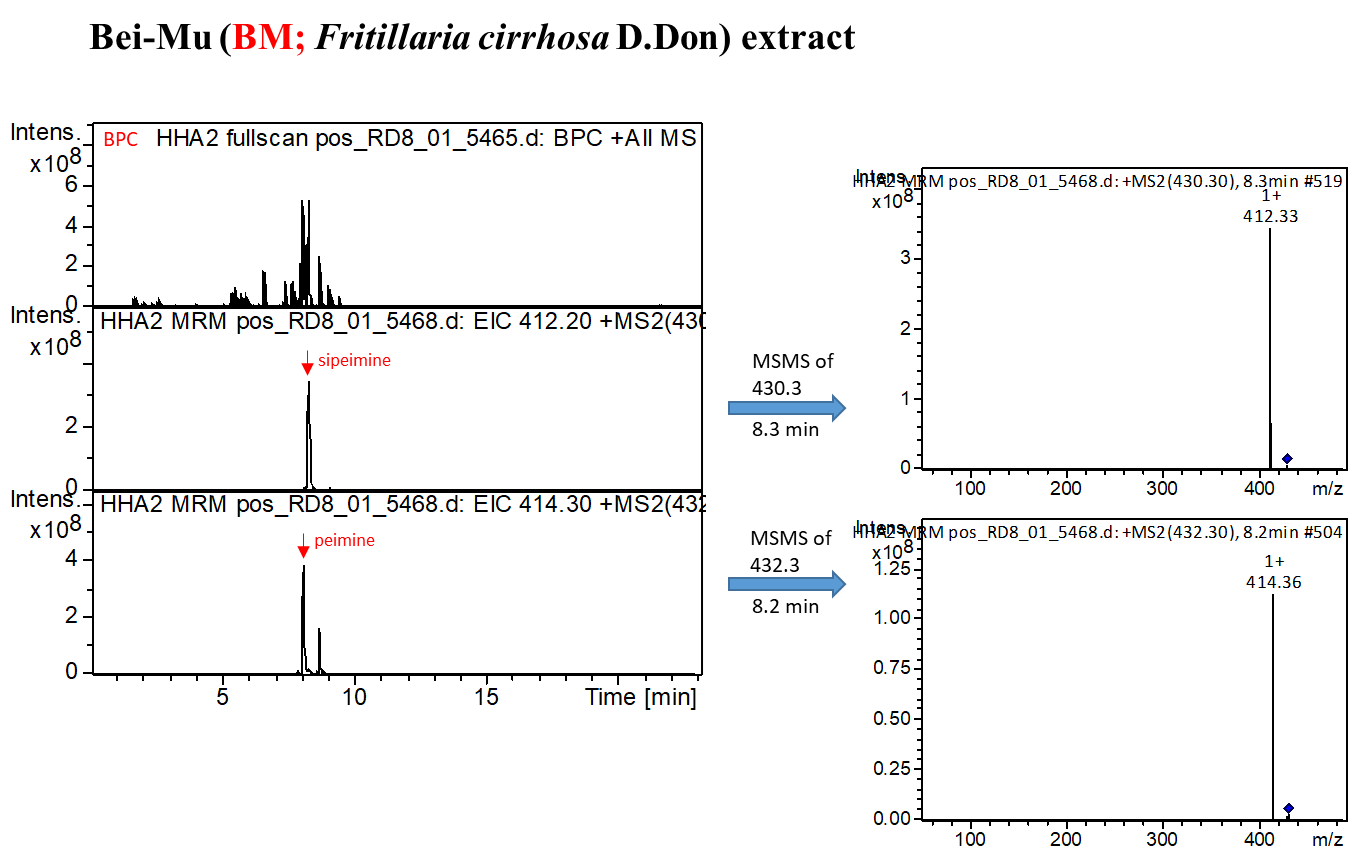
**

**Figure S5. A**


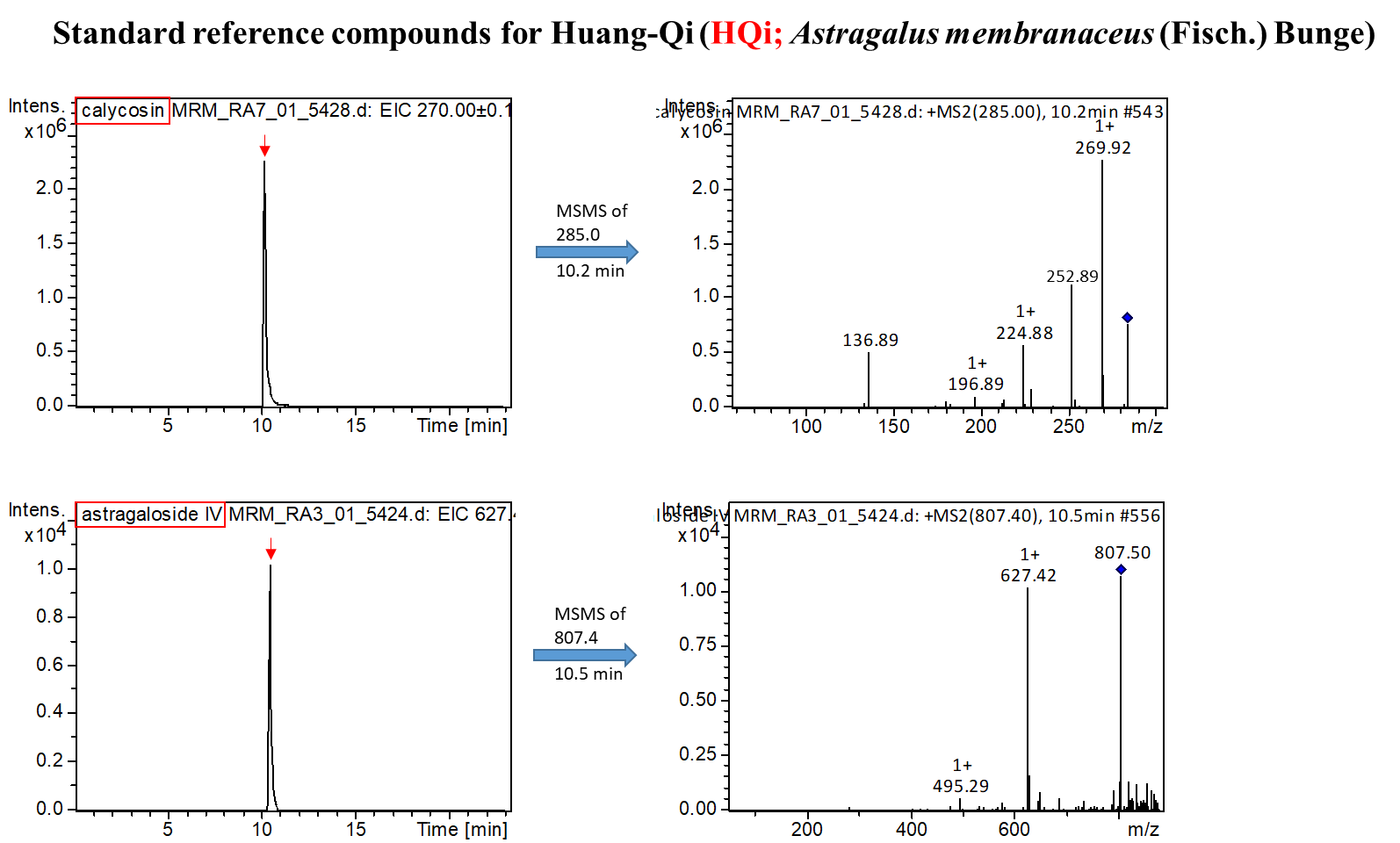


**Figure S5. B**


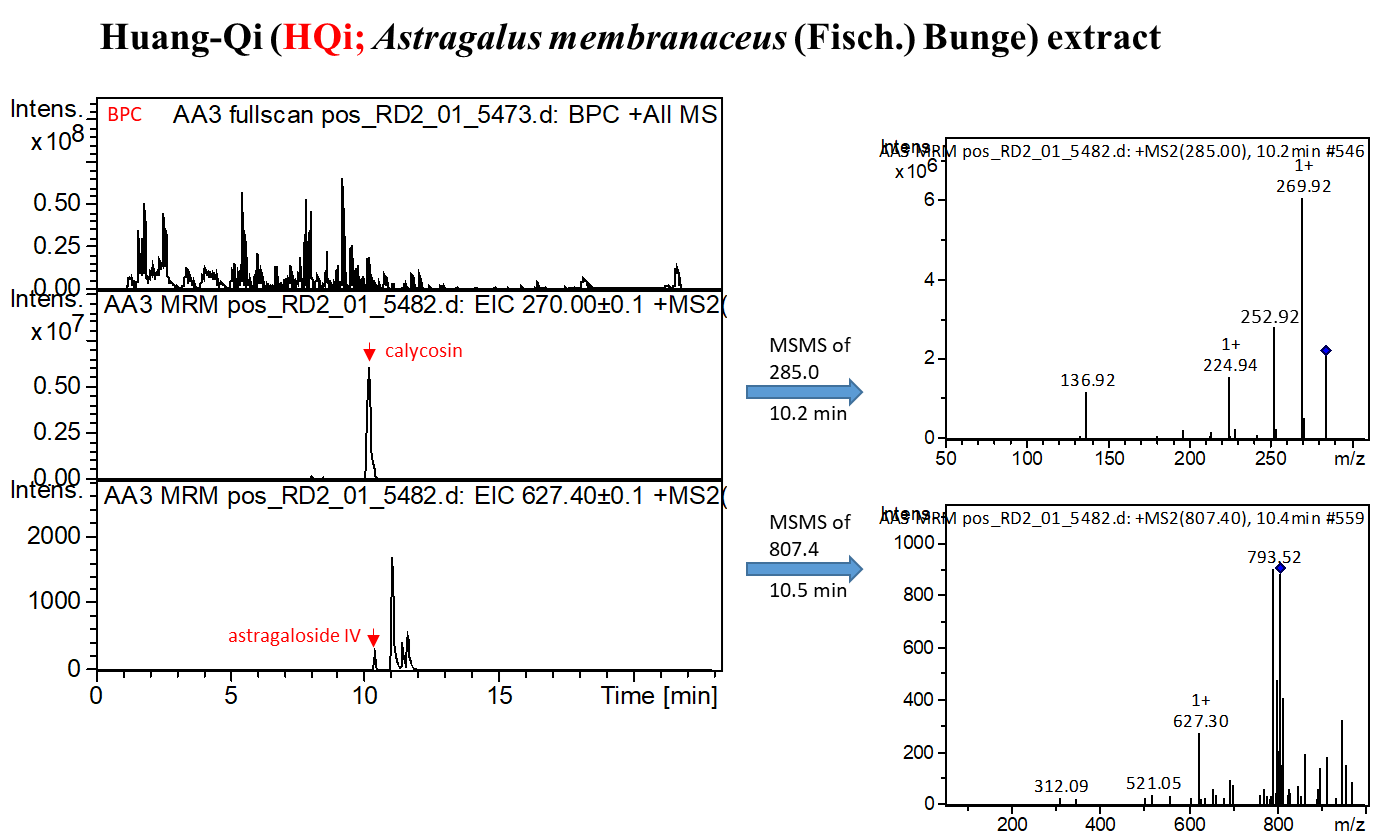


**Figure S6. A**


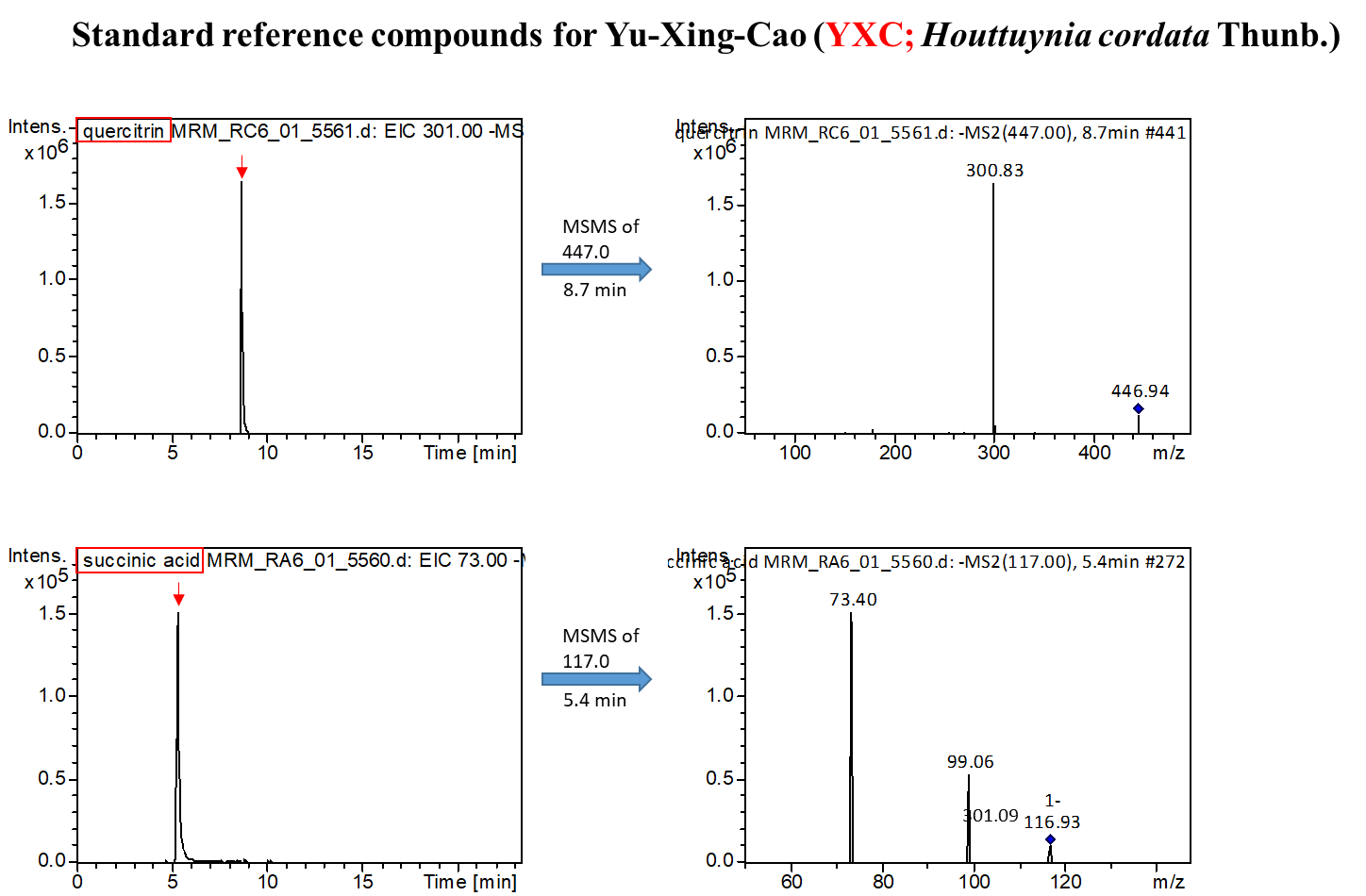


**Figure S6. B**

**
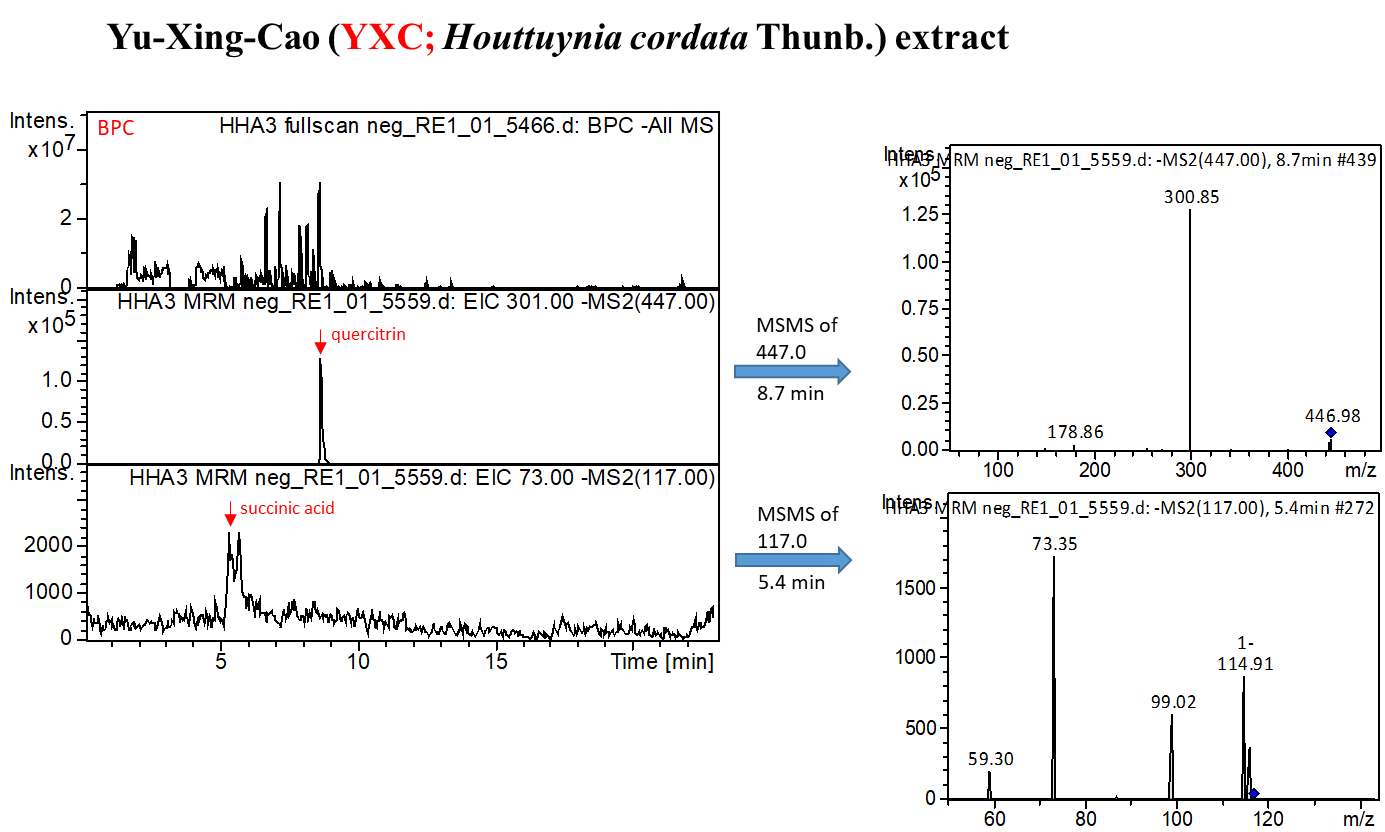
**

**Figure S7. A**


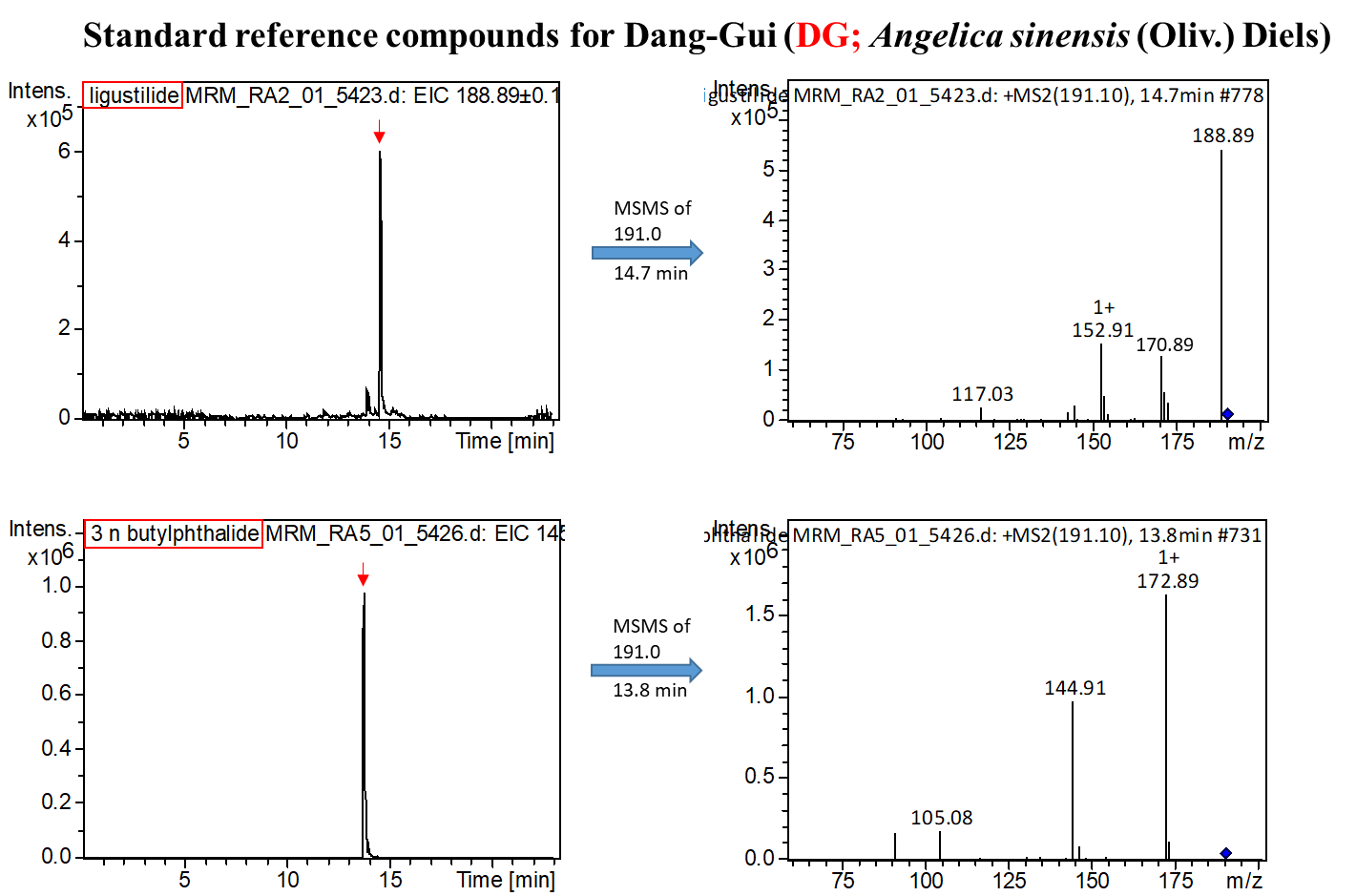


**Figure S7. B**


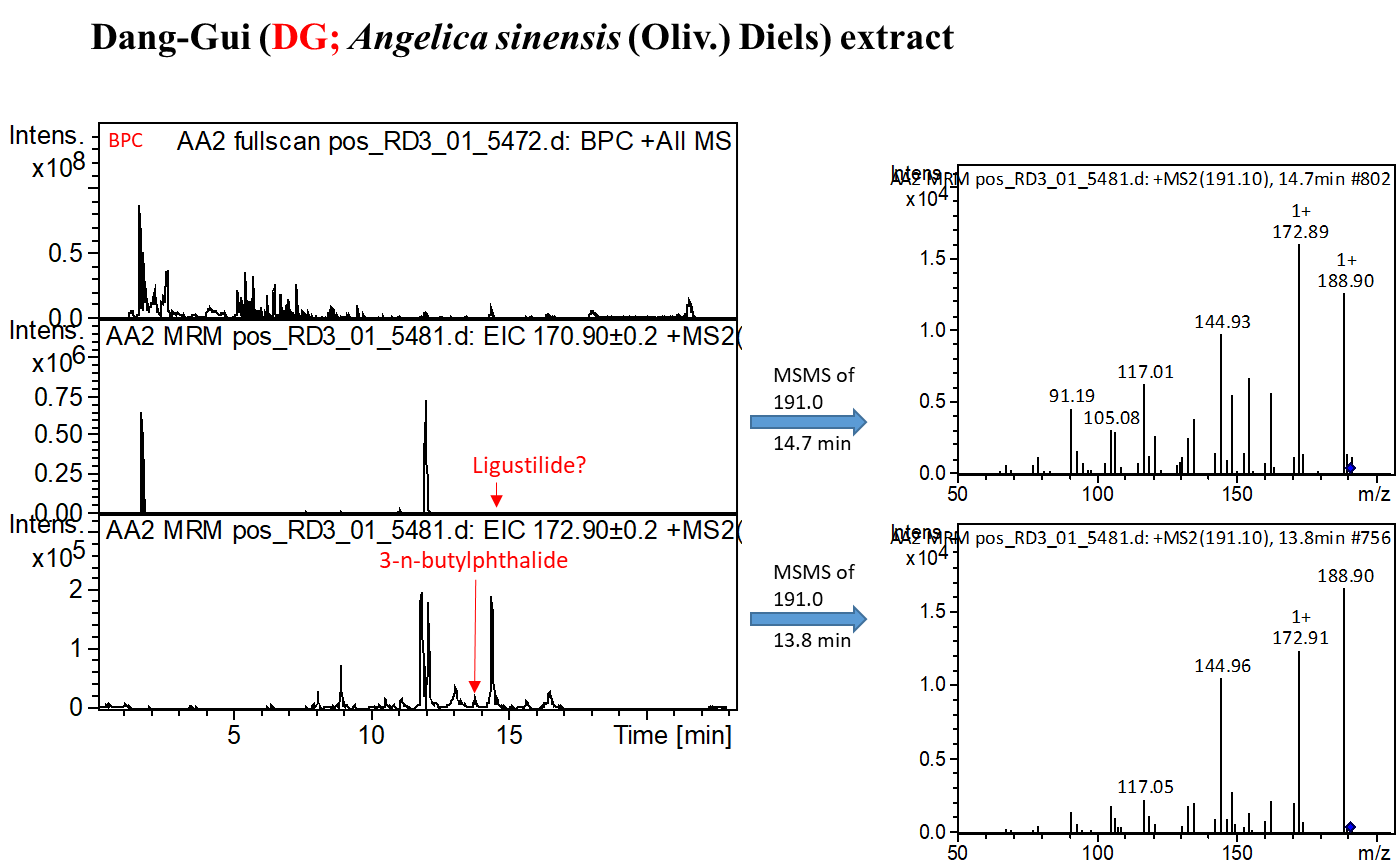

**Figure S8.**

| **TABLE S1 \| Composition of the most commonly used herbal formulas and single herbs for patients with hereditary hemolytic anemias in Taiwan** | | | | | | | | | | | | | |
| --- | --- | --- | --- | --- | --- | --- | --- | --- | --- | --- | --- | --- | --- |
| **Formulas** | **Chinese name** | **Total number of herbs** | **Number** | **Composition** | | | | **Therapeutic actions and indications** | **Frequency of prescriptions** | **Person-year** | **Percentage of usage person** | **Avg. drug dose per day (g)** | **Average duration for prescription (days)** |
|  |  |  |  | **Pin-yin name from American Dragon** | **Chinese name for single herb** | **Latin name** | **Botanical plant name** |  |  |  |  |  |  |
|  |  |  |  | **(http://www.americandragon.com/index.htm)** |  |  |  |  |  |  |  |  |  |
| **Total** |  |  |  |  |  |  |  |  | **378859** | **66252.70** | **100.00** | **12.69** | **7.57** |
| **Herbal formula (Pin-yin name)** |  |  |  |  |  |  |  |  | **368558** | **66155.30** | **99.74** | **9.37** | **7.56** |
| Xin-Yi-Qing-Fei-Tang (XYQFT) | 辛夷清肺湯 | 10 | 1 | Xin-Yi | 辛夷 | *Flos Magnoliae* | *Magnolia biondii* Pamp. | Antithrombotic and antioxidant activities and nourish blood deficiency (Pham et al., 2000; Zhang et al., 2013; Lin et al., 2019a; Liu et al., 2019b; Huang et al., 2006; Ku and Bae, 2014; Li et al., 2012) | 22590 | 27443.3 | 36.69 | 3.55 | 7.12 |
|  |  |  | 2 | Pi-Pa-Ye | 枇杷葉 | *Folium Eriobotryae* | *Eriobotrya japonica* (Thunb.) Lindl. |  |  |  |  |  |  |
|  |  |  | 3 | Zhi-Zi | 梔子 | *Fructus Gardeniae* | *Gardenia jasminoides* J.Ellis |  |  |  |  |  |  |
|  |  |  | 4 | Zhi-Mu | 知母 | *Rhizoma Anemarrhenae* | *Anemarrhena asphodeloides* Bge. |  |  |  |  |  |  |
|  |  |  | 5 | Bai-He | 百合 | *Bulbus Lilii* | *Lilium lancifolium* Thunb. |  |  |  |  |  |  |
|  |  |  | 6 | Huang-Qin | 黃芩 | *Radix Scutellariae* | *Scutellaria baicalensis* Georgi |  |  |  |  |  |  |
|  |  |  | 7 | Sheng-Ma | 升麻 | *Rhizoma Cimicifugae* | *Cimicifuga heracleifolia* Kom. |  |  |  |  |  |  |
|  |  |  | 8 | Mai-Men-Dong | 麥門冬 | *Radix Ophiopogonis* | *Ophiopogon japonicus* (Thunb.) Ker Gawl. |  |  |  |  |  |  |
|  |  |  | 9 | Shi-Gao | 石膏 | *Gypsum Fibrosum* | NA |  |  |  |  |  |  |
|  |  |  | 10 | Gan-Cao | 甘草 | *Radix Glycyrrhizae* | *Glycyrrhiza uralensis* Fisch. |  |  |  |  |  |  |
|  |  |  |  |  |  |  |  |  |  |  |  |  |  |
| **Single herbs (Pin-yin name)** |  |  |  |  |  |  |  |  | **314791** | **65080.90** | **97.94** | **4.27** | **7.67** |
| Gan-Cao (GC) | 甘草 | 1 | 1 | Gan-Cao | 甘草 | *Radix Glycyrrhizae* | *Glycyrrhiza uralensis* Fisch. | Antioxidant activity, nourish blood deficiency, and lowering the erythrocytic osmotic fragilitas (Lin et al., 2019a; Liu et al., 2019b; Lin et al., 1998) | 24870 | 27451.30 | 37.89 | 0.81 | 7.18 |
| Jie-Geng (JG) | 桔梗 | 1 | 1 | Jie-Geng | 桔梗 | *Radix Platycodi* | *Platycodon grandiflorus* (Jacq.) A.DC. | Antioxidant, anti-inflammatory, anti-cardiovascular diseases, anti-hypertension, anti-hyperlipidemia, and anti-diabetes activities (Lin et al., 2017; Fu et al., 2009; Nyakudya et al., 2014) | 22542 | 30016.90 | 41.21 | 1.01 | 6.66 |
| Bei-Mu (BM) | 貝母 | 1 | 1 | Bei-Mu | 貝母 | *Bulbus Fritillariae Cirrhosae* | *Fritillaria cirrhosa* D.Don | Analgesic, antioxidative, and anti-inflammatory effects (Chen et al., 2020b) | 22166 | 27869.80 | 38.22 | 1.02 | 7.41 |
| Huang-Qi (HQi) | 黃耆 | 1 | 1 | Huang-Qi | 黃耆 | *Radix Astragali* | *Astragalus membranaceus* (Fisch.) Bunge | Anti-anemia, promoting hematopoiesis activities (Huang et al., 2016; Liu et al., 2019a; Zhu and Zhu, 2001; Ren et al., 2016) | 18713 | 24930.20 | 35.10 | 1.40 | 9.10 |
| Yu-Xing-Cao (YXC) | 魚腥草 | 1 | 1 | Yu-Xing-Cao | 魚腥草 | *Herba Houttuyniae* | *Houttuynia cordata* Thunb. | Antioxidant, anti-inflammatory, and anti-diabetes activities (Kang et al., 2013;Kumar et al., 2014;Woranam et al., 2020) | 15185 | 22141.10 | 29.59 | 1.04 | 6.59 |
| Hai-Piao-Xiao (HPX) | 海螵蛸 | 1 | 1 | Hai-Piao-Xiao | 海螵蛸 | *Endoconcha Sepiae* | NA | Anti-stroke activity (Tsai et al., 2016) | 11232 | 12888.60 | 17.70 | 1.25 | 7.97 |
| Dang-Gui (DG) | 當歸 | 1 | 1 | Dang-Gui | 當歸 | *Radix Angelicae Sinensi* | *Angelica sinensis* (Oliv.) Diels | Anti-anemia, promoting hematopoiesis, and anti-inflammatory activities (Huang et al., 2016; Liu et al., 2019a; Wang et al., 2017; Chen et al., 2020c) | 9518 | 16315.40 | 22.46 | 1.05 | 9.09 |
| *Sorted by frequency of prescriptions. NA, not available. | | | | | | | | | | | | | |
| Information are obtained from the websites (http://www.americandragon.com/index.htm; http://old.tcmwiki.com/; http://www.shen-nong.com/eng/front/index.html; http://www.ipni.org/; http://www.theplantlist.org/). | | | | | | | | | | | | | |

| **TABLE S2 \| Frequency distribution of the cumulative CHM treatment days during the study period among patients with hereditary hemolytic anemias in Taiwan** | | | |
| --- | --- | --- | --- |
| **Cumulative CHM treatment days within the first year after hereditary hemolytic anemias** | **Cumulative CHM treatment days during the study period (started after the index date)** | **CHM users** | |
|  |  | **N** | **%** |
| **≥14 days (N = 9222)** |  |  | |
|  | **day < 180** | 4800 | 52.05 |
|  | **180 ≤ day< 360** | 1907 | 20.68 |
|  | **day ≥ 360** | 2515 | 27.27 |
| **≥28 days (N = 6328)** |  |  |  |
|  | **day < 180** | 2581 | 40.79 |
|  | **180 ≤ day< 360** | 1518 | 23.99 |
|  | **day ≥ 360** | 2229 | 35.22 |
| **≥56 days (N = 3743)** |  |  |  |
|  | **day < 180** | 979 | 26.16 |
|  | **180 ≤ day< 360** | 983 | 26.26 |
|  | **day ≥ 360** | 1781 | 47.58 |
| **≥84 days (N = 2446)** |  |  |  |
|  | **day < 180** | 409 | 16.72 |
|  | **180 ≤ day< 360** | 623 | 25.47 |
|  | **day ≥ 360** | 1414 | 57.81 |
| N, number; CHM, Chinese herbal medicine. | | | |
| The index date of this study was from the day on which the 14, 28, 56, or 84 cumulative days of CHM treatment with the first year were completed. | | | |
| Cumulative CHM treatment days during the study period was started after the index date. | | | |
